# A comprehensive simulation study evaluating the predictive performance of Cox proportional hazards model and machine learning methods for time-to-event data

**DOI:** 10.1186/s12874-026-03050-5

**Published:** 2026-11-10

**Authors:** Josline Adhiambo Otieno, Jenny Häggström, Marie Eriksson

**Affiliations:** Department of Statistics, USBE, Umeå University, Samhällsvetarhuset, Biblioteksgränd 6, Umeå, 901 87 Sweden

**Keywords:** Simulation, Machine learning, Survival analysis, Predictive modeling, Stroke

## Abstract

**Background:**

Many data-driven risk prediction models have been developed for analyzing time-to-event data. However, choosing the most suitable model for accurate predictions in a specific medical application remains a challenge. Simulation enables effective comparison on the basis of equal-sized datasets. This study provided a comprehensive and fair comparison of the overall survival prediction performance of machine learning models and the traditional Cox proportional hazards model, using both simulated and real datasets.

**Methods:**

Machine learning models included random survival forests, eXtreme Gradient Boosting, and deep neural networks (DeepSurv). We evaluated the performance of the models using the C-index and the Integrated Brier Score. The evaluation was performed under different data-generating mechanisms, such as varying sample sizes, censoring proportions, the addition of noise variables, and in the presence of different types of model misspecification.

**Results:**

All the models showed improved predictive performance with increasing sample sizes. However, their performance declined as the proportion of censoring increased. An increase in noise variables reduced prediction accuracy across all models, regardless of dataset size. Tree-based models demonstrated promising predictive performance compared to the Cox proportional hazards model and DeepSurv in the presence of misspecification and a large number of noise variables. The Cox proportional hazards model performed well with larger sample sizes and fewer noise variables. It also performed well when the model was correctly specified or had only minor misspecification.

**Conclusions:**

Appropriate model for a specific medical application should be motivated by the research question and the underlying data structure.

## Introduction

Recent developments in the medical field have shown that the ability to accurately diagnose disease and predict outcomes and implement optimal treatment strategies through data-driven risk prediction models is fundamental to improving healthcare delivery. In that regard, various risk prediction models based on machine learning (ML) have been developed. However, selecting the most accurate and reliable model for a specific medical application is challenging. For survival data applications, the well-known and celebrated Cox proportional hazards (PH) model is frequently adopted to model survival endpoints [1, 2]. Traditional approaches like the Cox PH model have the advantage of being simple to implement and understand; however, they rely on parametric or semiparametric distributions, require variable selection, and often face limitations when handling large or high-dimensional datasets where multicollinearity may exist [3]. ML methods have the potential to address these practical concerns by automatically capturing complex patterns and relationships in the data. The main drawback of the latter is its variation in interpretability depending on the complexity of the model [4].

Aside from the mentioned trade-offs, further methodological evaluation is required, as well as appropriate reporting on the development and validation of risk prediction models for their successful implementation in clinical practice [4, 5]. Researchers need to identify the best suited model for a given endpoint under different scenarios [6]. Furthermore, to enable a neutral comparison of ML models to the traditional ones, all models must be trained and evaluated on the same dataset, with similar splits and performance measures [7, 8]. A recent simulation study to determine the minimum sample size required to develop a reliable survival prediction model revealed that neural networks and random survival forests (RSF) require larger sample sizes to achieve performance comparable to that of traditional regression methods such as the Cox model [9].

Previous studies comparing conventional models with ML models to predict censored or survival data have studied a selection of these issues but have not extensively incorporated them [10–13]. A scoping review study identified simulation studies that compared statistical and ML models for the prediction of risk for survival outcomes, published between 2000 and 2020 [13]. In many of these studies, the data generation process, along with calculations for both the simulation estimands and performance metrics, was not clearly explained. Most of these studies only investigated a limited range of methods. Furthermore, while prior studies comparing Cox PH and ML survival models assess some relevant data-generating factors, these factors were generally not evaluated systematically or directly within a common framework (Table 1).

**Table 1:** Overview of simulation studies comparing statistical and machine learning models for survival prediction

Article	Model		Tuning strategy	Validation method	Metric	Characteristics of simulation design					Real-data application
	Statistical models	ML or Hybrid models				Sample size	Censoring proportion (%)	Noise variables	Mispecification		
									PH assumption	Nonlinearity	
Billichova et al. [14]	Cox	RSF, DeepSurv	Not clear	80/20 Train-test	C-index, IBS	250, 500, 2, 000, 6, 000	–	–	–	–	–
Birolo et al. [15]	Cox, EN-Cox	GrBSA, RSF, FastCPH, DeepHit, DSM, SurvTrace	Grid search	Nested CV	C-index, BS, ROC-AUC	300, 600, 1, 200, 2, 400	–	–	Yes	Yes	3 datasets
Graf et al. [16]	Cox	RSF	Grid search	. 632 + Bootstrap, Independent test set	C-index,IBS, Calibration curves	100, 200, 400	30, 60	–	Yes	–	2 RCT datasets
Kantidakis et al. [6]	Cox	PLANNs original/ extended	Grid search	5-fold CV	C-index, BS, IBS	250, 1, 000	20, 40, 61, 80	–	–	–	–
Infante et al. [9]	Cox, AFT ( ± splines)	Cox-boost, AFT-boost, RSF, PC-Hazard DNN	cvrisk, cvrisk, RF-SRC tune, Random search	10-fold CV, 10-fold CV, OOB obs., 3-fold CV	Brier score, IPA	600 − 9, 000 / 15,000	–	–	–	–	SEER registry data
Steingrimsson and Morrison [17]	Cox, Lasso-Cox	RSF, DeepSurv, CUDL	Default parameters, Partly, 5-fold CV for *η*	Independent simulated test data	MSPE, Brier score	1, 000, 250, 500, 1, 500, 3, 000	18, 47 (in different settings)	(Not explicitly reported)	Yes	–	Netherlands 70-gene data
Golmakani et al. [18]	Cox, Lasso-Cox, Ridge-Cox, EN-Cox	Cox-boost, Boosting, RSF	–, –, 1000 trees	10-fold CV	C-index	450, 720 (indirect)	–	30, 980 (indirect)	–	–	7 datasets
Wang and Li [19]	Lasso-Cox, Ridge-Cox	Cox-boost, RSF, NN	–, Default, –	2-fold CV	C-index, IBS	–	–	(not reported)	–	Yes	9 datasets
Katzman et al. [20]	Cox	RSF, DeepSurv	Not reported, Random search	Independent test set	C-index	–	–	(not varied)	–	Yes	5 datasets
Hu and Steingrimsson [21]	Cox, Lasso-Cox	RSF	Default	Independent test set	MSPE	200, 500	(not varied)	48,40 (in different settings)	Yes	–	German Breast Cancer
Gong et al. [22]	Cox	RSF, PLANN	Partly preset, Grid search	Independent test set	C-index	500, 200, 400, 600, 800, 1, 000	0, 25, 50, 75	(not varied)	–	Yes	–
Geng et al. [23]	Cox	SVM	Grid search	Independent test set	MSPE	100, 200	15,40	–	Yes	Yes	2 datasets
Lowsky et al. [24]	Cox	RSF, MKNN	Default, Grid search	Independent test set	IBS	500, 1, 000, 3, 000, 7, 500	–	0, 5, 10, 20, 30, 40	Yes	–	USRDSkidney-transplant
Omurlu et al. [25]	Cox	RSF	Default (1000 trees)	Bootstrap; OOB error	C-index	50, 100, 250,500	–	–	–	–	Breast cancer
Xiang et al. [26]	Cox	NN	Fixed	Repeated holdout	C-index	100, 200	0,20,30,50,70	–	Yes	–	–
**Our study**	**Cox**	**DeepSurv, RSF, XGBoost**	**Default**	**70/30 Train-test**	**C-index, IBS**	**500**, **1,000**, **5,000**, **10,000**	**0**, **10**, **50**, **70**, **85**	**0**, **15**, **100**, **500**	**Yes**	**Weak, Moderate**	**Riksstroke**

Despite the identified gaps in the existing studies, it is notable that they consistently applied a set of common ML and deep learning methods, including RSF, deep neural networks (DeepSurv) and survival gradient-boosting models [27, 28]. The latter reference suggested that both the RSF and DeepSurv models are currently the best performing models for predicting the outcomes of cardiovascular disease (CVD). This is in agreement with most of the findings from the application of ML in the prediction of survival outcome in oncology. For example, Xiao et al. revealed that the RSF model has a better discriminative ability compared to the support vector machine and the elastic net penalized Cox in predicting survival times of breast cancer patients [29], and RSF demonstrated better discrimination than the Cox regression model in predicting the overall survival probability of patients with laryngeal squamous cell carcinoma; however, both models had a similar calibration [30]. In addition, DeepSurv and RSF outperformed Cox-Time and DeepHit in terms of discrimination and calibration, respectively, in predicting disease-specific and overall survival of patients with oral cavity cancer [31].

Moving forward, data simulation is a powerful tool that can be adopted to help researchers evaluate existing approaches and understand the issues mentioned above. Since the data generation process is controlled, it enables a reliable evaluation of a model’s predictive performance [13]. As mentioned in the latter citation, simulation studies comparing statistical and ML models for prognostic modeling have not comprehensively or fairly evaluated these methods. The present study systematically compares the performance of the models across a broader range of data-generating conditions using a unified simulation design.

The primary objective is to evaluate and compare the survival prediction performance of three ML methods; eXtreme Gradient Boosting (XGBoost), RSF and DeepSurv, which are popular in the literature [13, 27, 28], to that of classical Cox regression, using both simulated and real datasets. We further analyze how the performance of these models varies under different data generating mechanisms including varying sample sizes, censoring proportions, number of noise variables, as well as the presence of different types of model misspecification, specifically, when the PH assumption is violated and when a quadratic covariate-effect model misspecification occurs. Furthermore, since few existing studies on predictive modeling of survival outcomes have focused on the application of ML methods in the CVD setting [11, 27, 32, 33], the present study evaluates and compares the predictive performance of these methods applied to survival after stroke based on data from the Swedish stroke registry (Riksstroke) [34].

The remainder of the paper is structured as follows. Section 2 details the methodological approach of this study, including a brief introduction to survival data analysis, the prediction models applied, the evaluation metrics used to assess model performance, a description of the simulation setting, followed by a description of the real survival data​set. Section 3 presents detailed analyses and summarizes the study findings based on different simulation settings and the real dataset. In Section 4, we discuss these findings in the context of the existing literature. Finally, a conclusion on the findings, summarizing key takeaways, and suggestions for relevant future studies are made in Section 5.

## Materials and methods

### Survival data analysis

In survival analysis, each observation (patient) *i* is associated with a tuple $\left\{\left({{\boldsymbol{x}}}_{i},{\delta }_{i},{t}_{i}\right);i=1,\ldots ,n\right\}$, where *n* is the total number of patients in the dataset, ${{\boldsymbol{x}}}_{i}=({x}_{i1},{x}_{i2},\cdots \,,{x}_{ip});{{\boldsymbol{x}}}_{i}\in {{\mathbb{R}}}^{1\times p}$ is a *p* − dimensional covariate vector for patient *i*, *δ*_*i*_ ∈ {0, 1} is a binary event indicator for patient *i*, satisfying *δ*_*i*_ = 0 if the event is censored and *δ*_*i*_ = 1 if the event occurred, and *t*_*i*_ is the observed event time defined as:

$${t}_{i}=\left\{\begin{array}{ll}{T}_{i}&\,\mathrm{if}\,\,{\delta }_{i}=1\\ {C}_{i}\,\mathrm{if}\,\,{\delta }_{i}=0\end{array}\right.,$$

where, *T*_*i*_ and *C*_*i*_ are nonnegative continuous random variables of event time and censoring time, respectively. For right censoring, *t*_*i*_ = min(*T*_*i*_, *C*_*i*_).

The primary focus of survival analysis is to estimate the conditional survival function given by the probability that the event of interest occurs after a specified time *t*, given covariates ***x***:

$$S(t| {\boldsymbol{x}})=P(T > t| {\boldsymbol{X}}={\boldsymbol{x}}).$$

This is conveniently estimated from the hazard function which describes the likelihood of the event occurring at time *t* given that no event has occurred before time *t*:

$$h(t| {\boldsymbol{x}})={\mathop{\lim }\limits_{\Delta t\to 0}}\dfrac{P(t\le T < t+\Delta t| T\ge t,{\boldsymbol{X}}={\boldsymbol{x}})}{\Delta t}.$$

The survival function can then be obtained as:

$$S(t| {\boldsymbol{x}})=\,\mathrm{exp}\,\left(-\int_{0}^{t}h(u| {\boldsymbol{x}})du\right).$$

### Prediction models

#### Cox proportional hazards (PH) model

Cox PH model is a survival analysis regression model that describes the association between the time until the occurrence of some event (the value of which can be censored for some patients) and a set of covariates. The outcome variable is expressed by the hazard function [1]. The model is semiparametric, that is, the distribution of both the outcome and the baseline hazard function is unknown, while the covariates are assumed to have an exponential influence on the outcome. It assumes linearity between the covariates and the log-hazard function. It refers to a PH model since the hazard rates between patients remain proportional over time.

Cox PH model assumes that the probability that a patient *i* survives beyond time *t*, given covariates ***x***_*i*_; *S*(*t*∣***x***_*i*_) = *P*(*T* > *t*∣***x***_*i*_) is defined by [1]:

1 $$h(t| {{\boldsymbol{x}}}_{i})={h}_{0}(t){e}^{{{\boldsymbol{x}}}_{i}{\boldsymbol{\beta }}},$$

where *h*(*t*∣***x***_*i*_) is the expected hazard at time *t* for patient *i*, *h*_0_(*t*) is a non-parametric baseline hazard function that represents the hazard of a patient when all covariates are set to their reference levels. ***x***_*i*_***β*** is referred to as the log-risk function of which ${\boldsymbol{\beta }}={({\beta }_{1},{\beta }_{2},\ldots ,{\beta }_{p})}^{\top }$ is a *p* × 1 column vector of coefficients of the respective *p* covariates, estimated from the data by optimizing the loss function (negative log-partial likelihood) of the Cox model given by:

2 $$\begin{array}{rcl}{\mathcal{L}}({\boldsymbol{\beta }})&=&\mathop{\prod }\nolimits_{i = 1}^{n}{\left[\dfrac{{e}^{{{\boldsymbol{x}}}_{i}{\boldsymbol{\beta }}}}{{\sum }_{j\in {\mathcal{R}}({t}_{i})}{e}^{{{\boldsymbol{x}}}_{j}{\boldsymbol{\beta }}}}\right]}^{{\delta }_{i}},\\ -\log {\mathcal{L}}({\boldsymbol{\beta }})&=&-\mathop{\sum }\limits_{i=1}^{n}{\delta }_{i}\left[{{\boldsymbol{x}}}_{i}{\boldsymbol{\beta }}-log\left(\sum _{j\in {\mathcal{R}}({t}_{i})}{e}^{{{\boldsymbol{x}}}_{j}{\boldsymbol{\beta }}}\right)\right],\end{array}$$

satisfying *δ*_*i*_ = 1, the *i*^*th*^ term in the product is the conditional probability, and for *δ*_*i*_ = 0, the *i*^*th*^ term equates to 0, which does not contribute to the final product. ${\mathcal{R}}({t}_{i})$ is the risk set at time *t*_*i*_ that includes all patients who are at risk (who have not experienced the event) just before the time when patient *i* experiences the event.

The optimal value of the risk scores ${e}^{{{\boldsymbol{x}}}_{i}{\boldsymbol{\beta }}}$ that relates to the survival time of patient *i* can then be calculated. *h*_0_(*t*) is also obtained from the data without pre-specifying its distribution. That is, it is indirectly estimated through the cumulative baseline hazard function, which is computed at each event time using the Breslow estimator:

$${\hat{H}}_{0}(t)=\sum _{{t}_{i}\le t}{\hat{h}}_{0}({t}_{i}),\,\,\,\mathrm{where}\,\,\,{\hat{h}}_{0}({t}_{i})=\dfrac{{d}_{i}}{{\sum }_{j\in {\mathcal{R}}({t}_{i})}{e}^{{{\boldsymbol{x}}}_{j}{\hat{\boldsymbol{\beta }}}}},$$

and *d*_*i*_ is the total number of events at time *t*_*i*_.

The survival probability until time *t* for each patient is finally calculated from the latter two estimates. That is, for a patient with covariates ***x***_*i*_, the survival probability is estimated as:

$$\hat{S}(t| {{\boldsymbol{x}}}_{i})={e}^{\left(-{\hat{H}}_{0}(t){e}^{{{\boldsymbol{x}}}_{i}{\hat{\boldsymbol{\beta }}}}\right)}.$$

#### Survival neural networks (DeepSurv)

The DeepSurv model is an extension of the classical Cox model which was proposed by Katzman et al. [20]. It is a Cox PH deep neural network developed for modeling complex relationships between a patient’s covariates and disease outcome. DeepSurv model learns the non-linear relationship between a patient’s risk factors and their risk of failure,the survival of laryngeal squamous cell carcinoma while the PH assumption of the model still holds. It is trained to model the log-risk function defined in Equation (1). The network consists of an input layer that takes ***x***_*i*_, hidden layers (fully connected layer of nodes), dropout layer, and an output layer that provides the estimated log-risk function parameterized by the weights of the network. To model the non-linearity in the data, the output of each layer is transformed by a non-linear activation function before it is passed to the next layer. A gradient descent algorithm is used to estimate the weights by minimizing the loss function of the model, which is based on the average loss function of the Cox model in Equation (2) defined by:

$$\begin{array}{lll}-\log {\mathcal{L}}({\boldsymbol{\theta }})=&-\dfrac{1}{\mathop{\sum }\nolimits_{i = 1}^{n}{\delta }_{i}}\left\{\mathop{\sum }\limits_{i=1}^{n}{\delta }_{i}\left[{{\boldsymbol{x}}}_{i}{\boldsymbol{\theta }}-log\left(\sum _{j\in {\mathcal{R}}({t}_{i})}{e}^{{{\boldsymbol{x}}}_{j}{\boldsymbol{\theta }}}\right)\right]\right\}\\&+\Psi \cdot \parallel {\boldsymbol{\theta }}{\parallel }_{2}^{2},\end{array}$$

where, $\Psi \cdot \parallel {\boldsymbol{\theta }}{\parallel }_{2}^{2}$ is the *L*^2^ regularization term that prevents overfitting, *Ψ* (*weight_decay*) is the regularization hyperparameter and ***θ*** is the *p* × 1 column vector of the weights of the neural net to be optimized. ${\boldsymbol{x}}{\hat{\boldsymbol{\theta }}}$ is the estimated log-risk function.

Similarly to the Cox regression approach (Section 2.2.1), the optimal estimate of risk scores ${e}^{{{\boldsymbol{x}}}_{i}{\boldsymbol{\theta }}}$ and survival probabilities at each event time point *t*_*i*_ until time *t*, for each patient *i* can then be obtained.

#### Random survival forests (RSF)

Random forests were originally proposed for the classification of dichotomous outcomes using tree-structured models [35], and have since been extended to the analysis of (right-censored) survival data [36–39].

RSF is a nonparametric ensemble of many survival trees that uses a splitting criterion to maximize the difference in survival probabilities between the resulting nodes. The algorithm starts by randomly generating *B* bootstrap samples with or without replacement from the original dataset, where each bootstrap sample (in-bag (IB)) leaves out approximately 37% of the original dataset. The omitted portion is referred to as out-of-bag data, and it can be used to evaluate the prediction performance of the model during training. For each bootstrap sample, the algorithm builds a decision tree by performing an exhaustive search through all the possible binary splits with the covariates and selects the best one following a splitting criterion. At each node, a random subset of covariates is used, usually the square root of the number of covariates, for splitting. Based on the splitting criterion, each of these candidate covariates is evaluated to determine one that maximizes the separation between the nodes, that is, maximizes the difference between the estimated survival curves in the resulting daughter nodes. The tree growing is stopped when a specific stop criterion is met, that is, when the terminal node has fewer than a predefined number of observations. In the final step, the cumulative hazard function (CHF) is calculated by averaging the Nelson-Aalen CHFs from the terminal nodes of each tree. The survival function for each patient is also obtained by averaging the Kaplan-Meier survival functions from the terminal nodes of each tree.

For each terminal node, the Nelson-Aalen estimator for CHF at time point *t*, which represents the expected number of events (cumulative hazard) per patient in leaf *k* by time *t*, is expressed as:

$$\hat{{H}_{k}}(t)=\sum _{{t}_{jk\le t}}\dfrac{{d}_{jk}}{{R}_{jk}},$$

while the survival function is formulated as:

$$\hat{{S}_{k}}(t)={\prod }_{{t}_{jk\le t}}\left(1-\dfrac{{d}_{jk}}{{R}_{jk}}\right),$$

where, *t*_*jk*_ is the *j*^*th*^ distinct event time among the patients in leaf *k*, *d*_*jk*_ and *R*_*jk*_ are the number of events and the total number of patients at risk at time *t*_*jk*_, respectively.

The aggregated CHF and survival function for the IB data can then be expressed respectively as follows:

$${\hat{H}}^{IB}(t| {{\boldsymbol{x}}}_{i})=\dfrac{\mathop{\sum }\nolimits_{b = 1}^{B}{I}_{ib}{\hat{H}}_{b}(t| {{\boldsymbol{x}}}_{i})}{\mathop{\sum }\nolimits_{b = 1}^{B}{I}_{ib}},$$

$${\hat{S}}^{IB}(t| {{\boldsymbol{x}}}_{i})=\dfrac{\mathop{\sum }\nolimits_{b = 1}^{B}{I}_{ib}{\hat{S}}_{b}(t| {{\boldsymbol{x}}}_{i})}{\mathop{\sum }\nolimits_{b = 1}^{B}{I}_{ib}},$$

satisfying *I*_*ib*_ = 1 when *i* is an IB case for *b*, otherwise it is set to 0. ${\hat{H}}_{b}(t| {{\boldsymbol{x}}}_{i})$ and ${\hat{S}}_{b}(t| {{\boldsymbol{x}}}_{i})$ respectively, are the CHF and survival function of the node in *b*^*th*^ bootstrap sample to which ***x***_*i*_ belongs.

The estimated average CHF and survival function of all *B* bootstrap samples for patient *i*, are then formulated, respectively, as:

$$\hat{H}(t| {{\boldsymbol{x}}}_{i})=\dfrac{1}{B}\mathop{\sum }\limits_{b=1}^{B}{\hat{H}}_{b}(t| {{\boldsymbol{x}}}_{i}),\quad \hat{S}(t| {{\boldsymbol{x}}}_{i})=\dfrac{1}{B}\mathop{\sum }\limits_{b=1}^{B}{\hat{S}}_{b}(t| {{\boldsymbol{x}}}_{i}).$$

It follows that the predicted risk score for the patient *i*, estimated at the prediction horizon *t*^⋆^, is given by:

$${\hat{r}}_{i}^{\,\mathrm{RSF}\,}({t}^{\star })=\hat{H}({t}^{\star }| {{\boldsymbol{x}}}_{i}).$$

#### eXtreme gradient boosting (XGBoost) for survival analysis

XGBoost is an optimized gradient boosting algorithm that is based on decision trees [40]. It is an ensemble learning algorithm which sequentially combines multiple weak classifiers (decision trees) to obtain a strong one. During the optimization process, the algorithm utilizes second-order gradient statistics to select optimal splits of tree nodes and to calculate the estimate of leaf nodes, while incorporating a regularization term to prevent overfitting. For application in survival analysis, the objective (loss) function of XGBoost was modified into the Cox model loss function to adapt to the time-to-event data. The general regularized learning objective function at the *m*^*th*^ iteration is given by:

$${{\mathcal{L}}}^{(m)}=\mathop{\sum }\limits_{i=1}^{n}l\left({t}_{i},{\hat{r}}_{i}^{(m-1)}+{f}_{m}({{\boldsymbol{x}}}_{i})\right)+\Omega ({f}_{m}),$$

where, ${\hat{r}}_{i}^{(m-1)}$ is the estimated log-risk function (predicted risk score) from the previous tree (iteration *m* − 1) for patient *i*, *f*_*m*_(***x***_*i*_) is the prediction of the current tree *f*_*m*_ (*m*^*th*^ decision tree), and $\Omega ({f}_{m})=\gamma {K}_{m}+\frac{1}{2}\eta \cdot \parallel w{\parallel }^{2}$ is the regularization term that controls the complexity of the tree *f*_*m*_. *γ* and *η* are the hyperparameters for regularization, *K*_*m*_ are the number of leaves in tree *f*_*m*_, and *w* is the vector of leaf weights.

Applying second-order gradient statistics to approximate the objective function:

$$\begin{array}{lll}{{\mathcal{L}}}^{(m)}\approx \mathop{\sum }\limits_{i=1}^{n}\left[l\left({t}_{i},{\hat{r}}_{i}^{(m-1)}\right)+{g}_{i}{f}_{m}({{\boldsymbol{x}}}_{i})+\dfrac{1}{2}{h}_{i}{f}_{m}^{2}({{\boldsymbol{x}}}_{i})\right]\\+\Omega ({f}_{m}),\end{array}$$

where ${g}_{i}=\frac{\partial l\left({t}_{i},{\hat{r}}_{i}^{(m-1)}\right)}{\partial {\hat{r}}_{i}^{(m-1)}}$ and ${h}_{i}=\frac{{\partial }^{2}l\left({t}_{i},{\hat{r}}_{i}^{(m-1)}\right)}{\partial {\hat{r}}_{i}^{(m-1)\cdot 2}}$ are, respectively, the first- and second-order gradients of the loss function with respect to the predictions at iteration *m*.

The final predicted risk score for patient *i*, ${\hat{r}}_{i}$ can then be obtained from the final prediction model that comprises an additive model of *M* trees as follows:

$${\hat{r}}_{i}=f({{\boldsymbol{x}}}_{i})=\mathop{\sum }\nolimits_{m = 1}^{M}{f}_{m}({{\boldsymbol{x}}}_{i}).$$

Similarly to the Cox PH model, the survival probabilities for patient *i* at each event time can also be estimated through the cumulative baseline hazard function, ${\hat{H}}_{0}(t)$, which is estimated by the Breslow estimator, as follows:

$$\hat{S}(t| {{\boldsymbol{x}}}_{i})={e}^{\left(-{\hat{H}}_{0}(t){e}^{f({{\boldsymbol{x}}}_{i})}\right)},\quad \,\mathrm{where}\,\quad {\hat{H}}_{0}(t)=\sum _{{t}_{i}\le t}\dfrac{{e}^{f({{\boldsymbol{x}}}_{i})}}{{\mathcal{R}}({t}_{i})}.$$

### Performance metrics

#### C-index

C-index is the most popular metric for evaluating the predictive accuracy of prognostic models in survival analysis. It measures how well the model discriminates between early and later events that are associated with high and low risk patients, respectively [41, 42]. Let (*t*_*i*_, *t*_*j*_) and (*r*_*i*_, *r*_*j*_) be a pair of observed survival times and predicted risk scores for any randomly selected pair of patients (*i*, *j*), respectively. For a good discriminative predictive model, the patient with the highest risk score will have a shorter survival time with a probability equal to 1. A C-index probability can be expressed as:

$$C-\,\mathrm{index}\,=P({r}_{i} > {r}_{j}\,| \,{t}_{i} < {t}_{j}).$$

C-index value ranges from 0 to 1, where a value of 1 corresponds to the best model and 0.5 corresponds to a random prediction.

#### Harrell’s C-index

Harrell’s approach is the most commonly used method for estimating the C-index in survival analysis [41, 42]. This is based on comparable pairs. A pair of patients (*i*, *j*) is comparable if we can determine which of them first experienced the event. A comparable pair is concordant (*c*) if the patient who experiences the event early is identified to have the higher risk and discordant (*d*) otherwise. Harrell’s C-index is defined as the ratio of the total number of concordant and comparable pairs as follows:

3 $$C-{\mathrm{index}}_{Harrell}=\dfrac{c+\dfrac{{t}_{r}}{2}}{c+d+{t}_{r}},$$

where, *c* is the count of pairs, which when both patients’ event times is observed; (*r*_*i*_ > *r*_*j*_∣*t*_*i*_ < *t*_*j*_)or(*r*_*i*_ < *r*_*j*_∣*t*_*i*_ > *t*_*j*_), when only patient *i* event is observed; (*r*_*i*_ > *r*_*j*_∣*t*_*i*_ < *t*_*j*_), and when only patient *j* event is observed; (*r*_*i*_ < *r*_*j*_∣*t*_*i*_ > *t*_*j*_), *d* is the count of all pairs in which, when both patients’ event times is observed; (*r*_*i*_ > *r*_*j*_∣*t*_*i*_ > *t*_*j*_)or(*r*_*i*_ < *r*_*j*_∣*t*_*i*_ < *t*_*j*_), when only patient *i* event is observed; (*r*_*i*_ < *r*_*j*_∣*t*_*i*_ < *t*_*j*_), and when only patient *j* event is observed; (*r*_*i*_ > *r*_*j*_∣*t*_*i*_ > *t*_*j*_), *t*_*r*_ is the total number of tied pairs on the risk scores. All other pairs are discarded from the construction of the index [41, 43]. Equation (3) can be comprehensively formulated as [42]:

$$\begin{array}{lll}C-{\mathrm{index}}_{Harrell}\\=\dfrac{\mathop{\sum }\nolimits_{i = 1}^{n}\mathop{\sum }\nolimits_{j = 1}^{n}\left\{{\mathbb{I}}\left({t}_{i} < {t}_{j}\right){\mathbb{I}}\left({r}_{i} > {r}_{j}\right){\delta }_{i}\right\}}{\mathop{\sum }\nolimits_{i = 1}^{n}\mathop{\sum }\nolimits_{j = 1}^{n}\left\{{\mathbb{I}}\left({t}_{i} < {t}_{j}\right){\delta }_{i}\right\}},\end{array}$$

where, ${\mathbb{I}}(\cdot)$ is an indicator function.

Although Harrell's C-index is commonly used to evaluate risk models in survival analysis, it tends to be overly optimistic and biased upward when the proportion of censoring in the dataset is large [44, 45]. Add61ition​ally, since it is a rank correlation measure, it is problematic to order survival times in the presence of censoring.

#### Uno's C-index

Uno’s C-index overcomes the bias introduced by censoring through the inverse probability of censoring weighting [44]. This is given by:

$$\begin{array}{lll}C-{\mathrm{index}}_{Uno}\\=\dfrac{\mathop{\sum }\nolimits_{i = 1}^{n}\mathop{\sum }\nolimits_{j = 1}^{n}\left\{{\mathbb{I}}\left({t}_{i} < {t}_{j}\right){\mathbb{I}}\left({r}_{i} > {r}_{j}\right){\left\{\hat{G}({t}_{i})\right\}}^{-2}{\delta }_{i}\right\}}{\mathop{\sum }\nolimits_{i = 1}^{n}\mathop{\sum }\nolimits_{j = 1}^{n}\left\{{\mathbb{I}}\left({t}_{i} < {t}_{j}\right){\left\{\hat{G}({t}_{i})\right\}}^{-2}{\delta }_{i}\right\}},\end{array}$$

where, $\hat{G}(\cdot)$ is the Kaplan-Meier estimator of the censoring time distribution.

#### Integrated bri​er score (IBS)

The (expected) Brier score (BS), originally introduced in 1950 for weather forecasting related to the probability of rain, is currently applied in survival analysis to assess how well a model predicts survival probabilities over time [46]. It measures the mean squared error between the observed survival status and the predicted survival probability of patient *i* at a fixed time point t^*^[47]. This can be expressed as:

4 $$BS({t}^{* })=\dfrac{1}{n}\mathop{\sum }\limits_{i}^{n}{\left({\mathbb{I}}({t}_{i}\ > \ {t}^{* })-\hat{S}({t}^{* }| {{\boldsymbol{x}}}_{i})\right)}^{2},$$

where, ${\mathbb{I}}({t}_{i} > {t}^{* })=1$ if the patient is still alive and had no event at time t^*^ otherwise equals 0 if the event occurred, and $\hat{S}({t}^{* }| {{\boldsymbol{x}}}_{i})$ is the predicted survival probability of the same patient at time t^*^ given covariates ***x***_*i*_, satisfying $0\le \hat{S}({t}^{* }| {{\boldsymbol{x}}}_{i})\le 1$ for *i* = 1, 2, 3, ⋯  , *n* patients.

Since BS depends on the user’s specified time point, comparison between models becomes challenging when one is interested in the average prediction performance over entire time interval. The integrated Brier score (IBS) is an evaluation metric that averages the BS over the entire range of observed survival times. Hence, it provides more information compared to BS. Building on Equation (4), this is formulated as:

5 $$IBS=\dfrac{1}{\,\mathrm{max}({t}_{i})}\int_{0}^{\mathrm{max}\,({t}_{i})}BS({t}^{* })dt.$$

Accounting for censoring through inverse probability censoring weighting (IPCW) [47, 48], Equation (5) becomes:

6 $$IBS=\left\{\begin{array}{l}\dfrac{1}{\,\mathrm{max}({t}_{i})}\int_{0}^{\mathrm{max}\,({t}_{i})}\left(\dfrac{1}{n}\mathop{\sum }\nolimits_{i}^{n}{\hat{w}}_{i}({t}^{* }){\left({\mathbb{I}}({t}_{i}\ > \ {t}^{* })-\hat{S}({t}^{* }| {{\boldsymbol{x}}}_{i})\right)}^{2}\right)dt\quad \\ \dfrac{1}{\,\mathrm{max}({t}_{i})}\int_{0}^{\mathrm{max}\,({t}_{i})}\dfrac{1}{n}\mathop{\sum }\nolimits_{i = 1}^{n}\left(\dfrac{{\left(\hat{S}({t}^{* }| {{\boldsymbol{x}}}_{i})\right)}^{2}}{\hat{G}({t}^{* }| {{\boldsymbol{x}}}_{i})}\cdot {\mathbb{I}}({t}_{i} > {t}^{* })\,+\right.\quad \\ \hspace{0.5em}\quad \quad \quad \quad \quad \quad \quad \quad \quad \left.\dfrac{{\left(1-\hat{S}({t}^{* }| {{\boldsymbol{x}}}_{i})\right)}^{2}}{\hat{G}({t}_{i}| {{\boldsymbol{x}}}_{i})}\cdot {\mathbb{I}}({t}_{i}\le {t}^{* })\cdot {\delta }_{i}\right)dt\quad \end{array}\right.,$$

where, ${\hat{w}}_{i}({t}^{* })=\frac{{\mathbb{I}}({t}_{i}\le {t}^{* })\cdot {\delta }_{i}}{\hat{G}({t}_{i}| {{\boldsymbol{x}}}_{i})}+\frac{{\mathbb{I}}({t}_{i} > {t}^{* })}{\hat{G}({t}^{* }| {{\boldsymbol{x}}}_{i})}$ is the inverse probability of censoring weights, $\hat{G}({t}^{* }| {{\boldsymbol{x}}}_{i})$ is the probability of being uncensored at time t^*^ given covariates ***x***_*i*_, and $\hat{G}({t}_{i}| {{\boldsymbol{x}}}_{i})$ is the probability of being uncensored at the observed time *t*_*i*_ given covariates ***x***_*i*_. The latter are both estimated by the Kaplan-Meier estimator of the censoring distribution.

The IBS values range between 0 and 1, where 0 indicates a perfect prediction model, while 1 means the worst possible predictions. Thus, a lower IBS shows a better performance of the prediction model.

### Simulation of survival data

Data were simulated with a censored survival time and a set of covariates to assess the performance of different ML models compared to the classical Cox PH model in predicting the overall survival time of patients after stroke. This study was conducted following the guidelines for healthcare simulation research [49, 50].

In an attempt to capture some aspects of the structure of the real data from Riksstroke, the datasets were generated with a set of 17 covariates (${{\boldsymbol{x}}}_{i}\in {{\mathbb{R}}}^{1\times 17};\,i=1,\cdots \,,n$ patients) and with varying functional relationships to survival time. To create a simplified and controlled simulation setting for model comparison, these covariates were independently sampled from a predefined distribution with specified parameters (more details are provided in Table A1 of Appendix A). The continuous covariate was drawn from a normal distribution, while the binary and categorical covariates were drawn from binomial and multinomial distributions, respectively.

Survival times (*T*) that follow an exponential distribution with a constant baseline hazard rate *λ* = 0.125, were generated using the Bender et al. formulae [51], which shows a general relation between hazard and survival time of the Cox PH model. This is defined as:

$$T=-\dfrac{\,\mathrm{log}\,(U)}{{h}_{0}(t){e}^{{{\boldsymbol{x}}}_{i}{\boldsymbol{\beta }}}}\,;\,\,U={e}^{-{H}_{0}(t){e}^{{{\boldsymbol{x}}}_{i}{\boldsymbol{\beta }}}}\,;\,\,{H}_{0}(t)=\int_{0}^{t}{h}_{0}(u)du,$$

where *H*_0_(*t*) is the cumulative baseline hazard function and *h*_0_(*t*) = *λ* is the baseline hazard function, which was assumed to be constant over time. *U* is sampled from the standard uniform distribution, *U*(0, 1). The covariates were combined through a specified functional form *η* = *f*(***x***_***i***_) to allow for both linear (*η* = ***x***_*i*_***β***) and non-linear effects of the covariates on survival time. The regression coefficients estimated from a Cox PH model fitted to the Riksstroke dataset were used as ***β*** for the main linear effects.

Censoring times were also simulated assuming an exponential distribution (*C* ~ Exponential(*α*)) and was independent of any covariate. In order to achieve a predefined censoring proportion, we set different values of the rate parameter *α*.

The survival outcome, including both the event and the censored times, was then generated by the minimum of the survival and censoring time.

We studied the influence of a number of factors on the predictive performance of the models for each of the data generating processes; including varying sample sizes (500, 1000, 5000, and 10000), changing the proportion of censoring (0%, 10%, 50% and 85%), adding noise covariates in the fitted model (0, 15, 100 and 500), and in the presence of different types of model misspecification, that is, assuming an incorrect functional form for the link between the hazard function and the covariates, as well as if the PH assumption is violated. Continuous noise covariates were sampled from a standard normal distribution, while binary noise covariates were sampled from a binomial distribution with probability 0.5.

Table 2 gives information on the different misspecification settings used during the simulation. The scenarios included the presence of weak non-linearity (a quadratic effect of 0.1 for the continuous predictor), moderate non-linearity (a quadratic effect of 0.9 for the continuous predictor), and when the PH assumption is violated. A deviation from the PH assumption was introduced by changing the effect of *x*_*i*3_ covariate from 0 to 3 at time 0.25, a time that corresponds to the median survival time in Riksstroke.

**Table 2:** Data generation model misspecification scenarios

Scenario	Model formulation		Specification
	Data generation model	Fitted model	
Weak non-linearity	$\mathop{\sum }\limits_{j=1}^{17}{\beta }_{j}{x}_{ij}+0.1{x}_{i1}^{2}$	$\mathop{\sum }\limits_{j=1}^{17}{\beta }_{j}{x}_{ij}+{\beta }_{k}{x}_{i1}^{2}$	Correct
	$\mathop{\sum }\limits_{j=1}^{17}{\beta }_{j}{x}_{ij}+0.1{x}_{i1}^{2}$	$\mathop{\sum }\limits_{j=1}^{17}{\beta }_{j}{x}_{ij}$	Incorrect
Moderate non-linearity	$\mathop{\sum }\limits_{j=1}^{17}{\beta }_{j}{x}_{ij}+0.9{x}_{i1}^{2}$	$\mathop{\sum }\limits_{j=1}^{17}{\beta }_{j}{x}_{ij}+{\beta }_{k}{x}_{i1}^{2}$	Correct
	$\mathop{\sum }\limits_{j=1}^{17}{\beta }_{j}{x}_{ij}+0.9{x}_{i1}^{2}$	$\mathop{\sum }\limits_{j=1}^{17}{\beta }_{j}{x}_{ij}$	Incorrect
Violated PH assumption	$$\mathop{\sum }\limits_{\begin{array}{c}j=1\\ j\ne 3\end{array}}^{17}{\beta }_{j}{x}_{ij}+{\beta }_{3}(t){x}_{i3}$$;$${\beta }_{3}(t)=\left\{\begin{array}{ll}0\quad &t < 0.25\\ 3\quad &\,\mathrm{otherwise.}\,\end{array}\right.$$	$$\mathop{\sum }\limits_{\begin{array}{c}j=1\\ j\ne 3\end{array}}^{17}{\beta }_{j}{x}_{ij}+{\beta }_{3}(t){x}_{i3}$$*β*_3_(*t*)*x*_*i*3_ ⇒ *x*_*i*3_ + (*x*_*i*3_*t > 0.25)	Correct
	$$\mathop{\sum }\limits_{\begin{array}{c}j=1\\ j\ne 3\end{array}}^{17}{\beta }_{j}{x}_{ij}+{\beta }_{3}(t){x}_{i3}$$;$${\beta }_{3}(t)=\left\{\begin{array}{ll}0\quad &t < 0.25\\ 3\quad &\,\mathrm{otherwise.}\,\end{array}\right.$$	$\mathop{\sum }\limits_{j=1}^{17}{\beta }_{j}{x}_{ij}$	Incorrect

The number of replications per data generation process in the simulation was set to 1000. All analyses were conducted in the *R* programming language v.4.2. 1 [52].

After generating each dataset, we trained the models and evaluated their predictive performance. Cox PH model was fitted using the coxph function of the survival package (v.3.5. 8). The correctly specified Cox model was fitted, allowing the *x*_*i*3_ effect to vary at time 0.25, using a counting process approach and a time-varying transformation. This was implemented by the survSplit function and the time-transform functionality of coxph from the survival package (v.3.5. 8) [53, 54]. DeepSurv was trained using the deepsurv function of the survivalmodels package (v.0.1. 191). XGBoost was implemented using the xgboost function of the xgboost package (v.1.7. 7.1). To train the RSF, we opted for the classical log-rank test statistics split criterion specified in the randomForestSRC package (v.3.2. 3) to select optimal splitting covariates, as the data consisted primarily of binary covariates with only one continuous covariate, minimizing the risk of selection bias [36, 55].

Stratified random sampling was used to randomly split each simulated dataset into two sets (70% train and 30% test set), maintaining the same proportion of censoring. To ensure consistency and comparability, all models were trained on the entire training dataset with default hyperparameters (more details are provided in Table D7 of Appendix D). The performance of the final models was then assessed on the test dataset based on Harrell’s C-index, Uno’s C-index, and IBS. To account for ties, the concordance function of the survival package (v.3.5. 8) was used to estimate both C-indexes. Regarding the computation of IBS values, we implemented Equation (6) through the brier function in the pec package (v.2023.4. 12), with integration performed over the observed follow-up time range. The correctly specified Cox model was evaluated using Harrell’s C-index, as there is currently no implementation of both Uno’s C-index and IBS for the counting process format for survival data.

### Real survival data

To evaluate predictive performance in a practical context, we also performed an analysis of survival after an ischemic stroke using data from Riksstroke[34]. Data included patients with ischemic stroke from 2015 to 2020. Patients aged between 18 and 108 years and were followed up from the onset of stroke until the time of death from any cause or the end of follow-up (censored), December 31, 2020. Therefore, the outcome of interest was overall survival, with predictions evaluated at the maximum observed follow-up time.

A total of 17 covariates including patient’s demographic characteristics such as age in years, sex, other cardiovascular risk factors including smoking, atrial fibrillation, diabetes, previous stroke, hypertension, stroke management (intravenous thrombolysis, endovascular thrombectomy, admission at stroke unit), secondary prevention (anticoagulants, lipid lowering medication), functional status (modified Rankin Scale (mRS)) prior to the stroke (points 0 − 2, 3, 4, 5), and stroke characteristics (loss of consciousness (alert, drowsy, unconscious), wake-up stroke, stroke alert activation, arrival at the hospital by ambulance), were retrieved (Table C6 of Appendix C). Since loss of consciousness was multinomial, one-hot encoding was performed to transform it into binary type suitable for regression analysis. To avoid perfect multicollinearity in the prediction models, the lowest category of this variable (alert) was omitted and used as the reference category. The mRS was classified into a binary covariate; independent (points 0 − 2) and dependent (points 3 − 5). This amounted to a total of 18 regression coefficients. Additionally, age was rescaled to have a mean of 0 and a standard deviation of 1.

This resulted in a total of 70289 cases with complete information and a censoring rate of 72% (19374 observed number of deaths within the 2191 days follow-up time). A total of 49202 (70%) cases were selected for training, while the remaining 21087 (30%) cases were used for model evaluation, ensuring that the censoring proportions were maintained in both sets. To allow a meaningful comparison between the results from Riksstroke and those generated by the simulation, analysis was also performed on sub-samples of 500 and 10000 ischemic stroke patients from the dataset described above.

### Hyperparameter tuning

Default settings were used for all ML models in the simulation study. Tuning these models in each of the 1, 000 replications across all simulation scenarios required a computational workload that could not be managed without access to high-performance computing, to which we did not have access. While using default hyperparameters simplifies the training process and reduces computational cost, it might result in suboptimal prediction performance. Therefore, we performed a hyperparameter optimization on the ML models using the sub-samples from Riksstroke, to observe the differences between the optimized and unoptimized models. We defined a range of hyperparameters to tune, as provided in Table 3. We then used Autotuner from the mlr3tuning package v.1.3. 0 to train and optimize the learner (from the ml3extralearners package v.1.0. 0) of each model. These included survival DeepSurv learner for DeepSurv, survival random forest SRC learner for RSF, and XGBoost Cox survival learner for XGBoost model. For each model, we used a 5-fold cross-validation on training data, optimized for the C-index, and used a Random Search strategy with 100 iterations.

**Table 3:** Model hyperparameters search space and optimized parameters from random search tuning, based on 2015 − 2020Riksstroke data set (ischemic stroke patients)

Hyperparameter	Search space	Optimized parameters	
		Sample size: 10000	Sample size: 500
**DeepSurv**			
$$\,\mathrm{num}\mathrm{\_}\mathrm{nodes}\,\left\{\begin{array}{l}\,\mathrm{num}\mathrm{\_}\mathrm{layers}\,\\ \,\mathrm{num}\mathrm{\_}\mathrm{nodes}\mathrm{\_}\mathrm{per}\mathrm{\_}\mathrm{layer}\,\end{array}\right.$$	[5, 20]	20	12
	[10, 50]	15	35
dropout	[0, 0.5]	0.212	0.086
weight_decay	[0, 0.5]	0.079	0.227
learning_rate	[0, 0.05]	0.030	0.042
activation	*c*(*”relu”*, *”sigmoid”*)	*”relu”*	*”relu”*
epochs	[1, 500]	13	387
**XGBoost**			
alpha	[0, 10]	9.652	7.552
subsample	[0.5, 1]	0.973	0.574
nrounds	[50, 1000]	540	158
colsample_bytree	[0, 1]	0.145	0.044
max_depth	[3, 40]	23	38
eta	[0.0001, 1]	0.054	0.313
gamma	[0.0001, 1]	0.666	0.468
lambda	[0, 10]	9.570	1.717
min_child_weight	[1, 10]	3	2
**RSF**			
nsplit	[10, 20]	16	19
ntree	[400, 1000]	689	551
mtry	[1, 20]	3	3
nodesize	[5, 20]	19	9
nodedepth	[10, 100]	12	59

## Results

### Simulation results

This section presents the results of the analyses conducted on simulated datasets, focusing on comparing the performance of the Cox PH model, DeepSurv, and tree-based models (RSF, XGBoost) in predicting overall survival time after stroke. The prediction performance of the models was evaluated at the maximum observed survival time generated in each simulation.

#### Variation in sample sizes

**Fig. 1:**
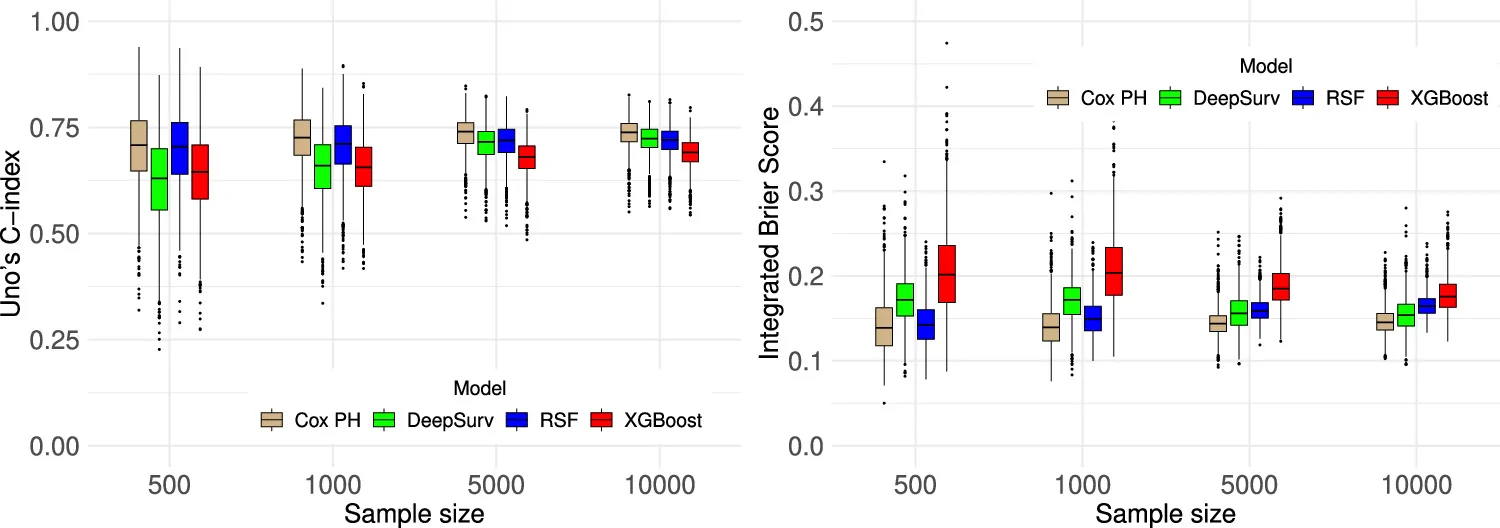
Predictive performance for varying sample sizes with censoring proportion of 85%

Figure 1 shows the performance of three ML models compared to the Cox PH model across different sample sizes in predicting overall survival time of patients (Table B2 of Appendix B1). The simulated dataset had an 85% censoring proportion. As indicated by Uno’s C-index, all models showed an improvement in performance with increasing sample sizes. However, the overlapping inter-quartile ranges (Table B2; confidence intervals) indicate no significant performance difference among these models. We also observed that the confidence intervals of the metric estimates narrow with increasing sample size, reflecting greater precision in the estimates when the sample size increases. The findings indicate that the Cox PH model shows consistently promising predictive performance across all sample sizes compared to the other models.

#### Varying censoring proportions

**Fig. 2:**
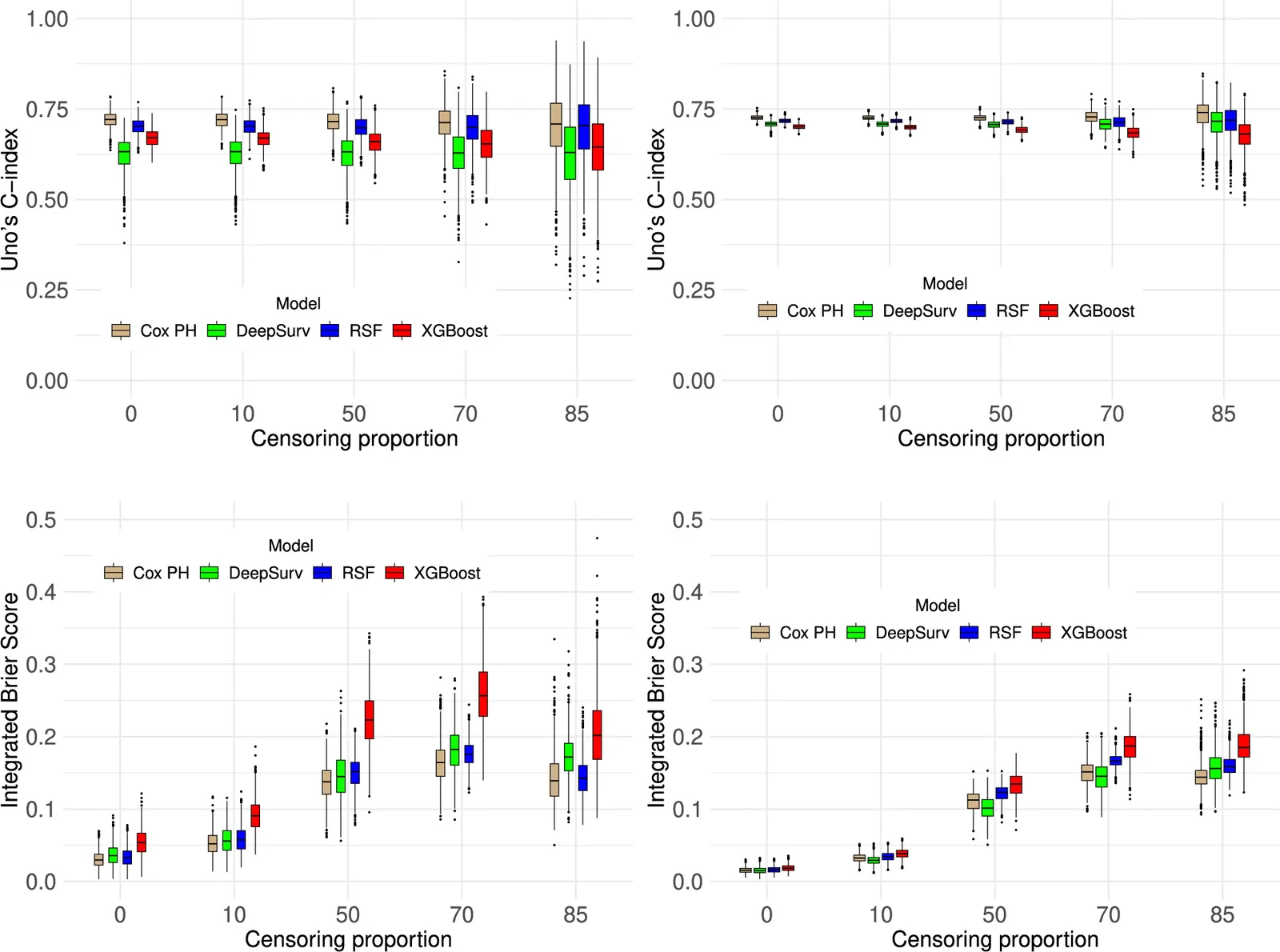
Prediction performance of the models for varying censoring proportions with sample sizes 500 (left) and 5000 (right)

The simulation results for model accuracy with various levels of censoring (0, 10, 50, 70, 85%) are displayed in Fig. 2 (also in Table B3 of Appendix B2). Uno’s C-index indicates that there is no notable change in the model’s prediction performance as the censoring proportion increases. However, the precision of the performance metrics estimates decreases as the proportion of censoring increases, which is demonstrated by the widening of the inter-quartile ranges (Table B3; confidence intervals), regardless of the sample size. IBS estimates indicate a decline in model prediction performances as the proportion of censoring increases. Even with a decline in predictive performance, the Cox PH model consistently demonstrates higher predictive accuracy compared to the other models across all censoring levels based on Uno’s C-index.

#### Adding noise variables

**Fig. 3:**
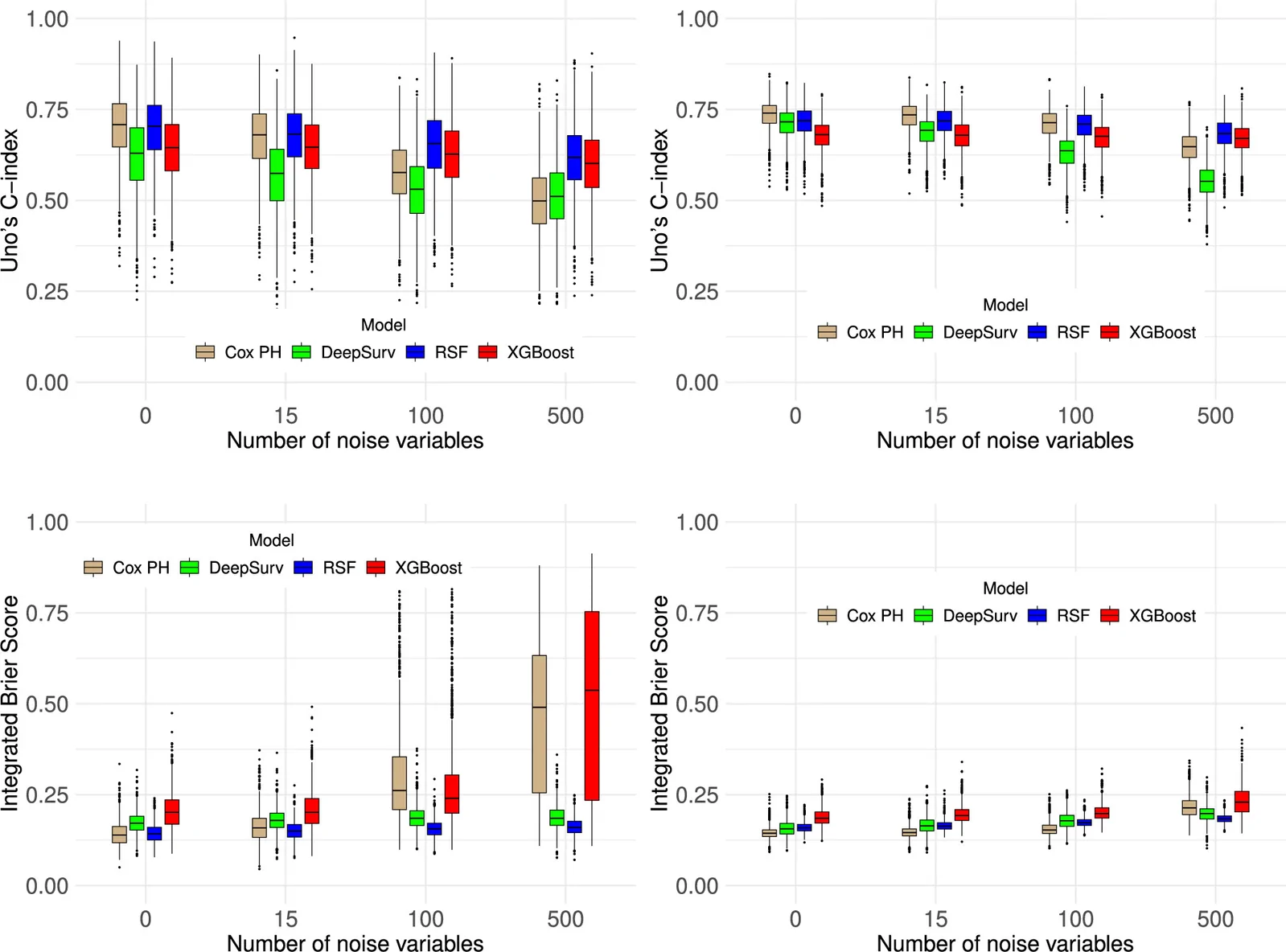
Prediction performance of the models for varying number of noise variables with censoring proportion of 85% and sample sizes of 500 (left) and 5000 (right)

The effect of adding a different number of noise variables (0, 15, 100, 500) to the prediction models is presented in Fig. 3 and Table B4 of Appendix B3. The results indicate that an increase in the number of noise variables leads to a decrease in prediction accuracy across all models, regardless of the sample size. As the number of noise variables increases, tree-based models demonstrate a modest improvement in prediction performance compared to the Cox PH model and DeepSurv. However, for a large sample size (*n* = 5000), the Cox PH model shows performance comparable to that of tree-based models.

#### Model misspecification

**Fig. 4:**
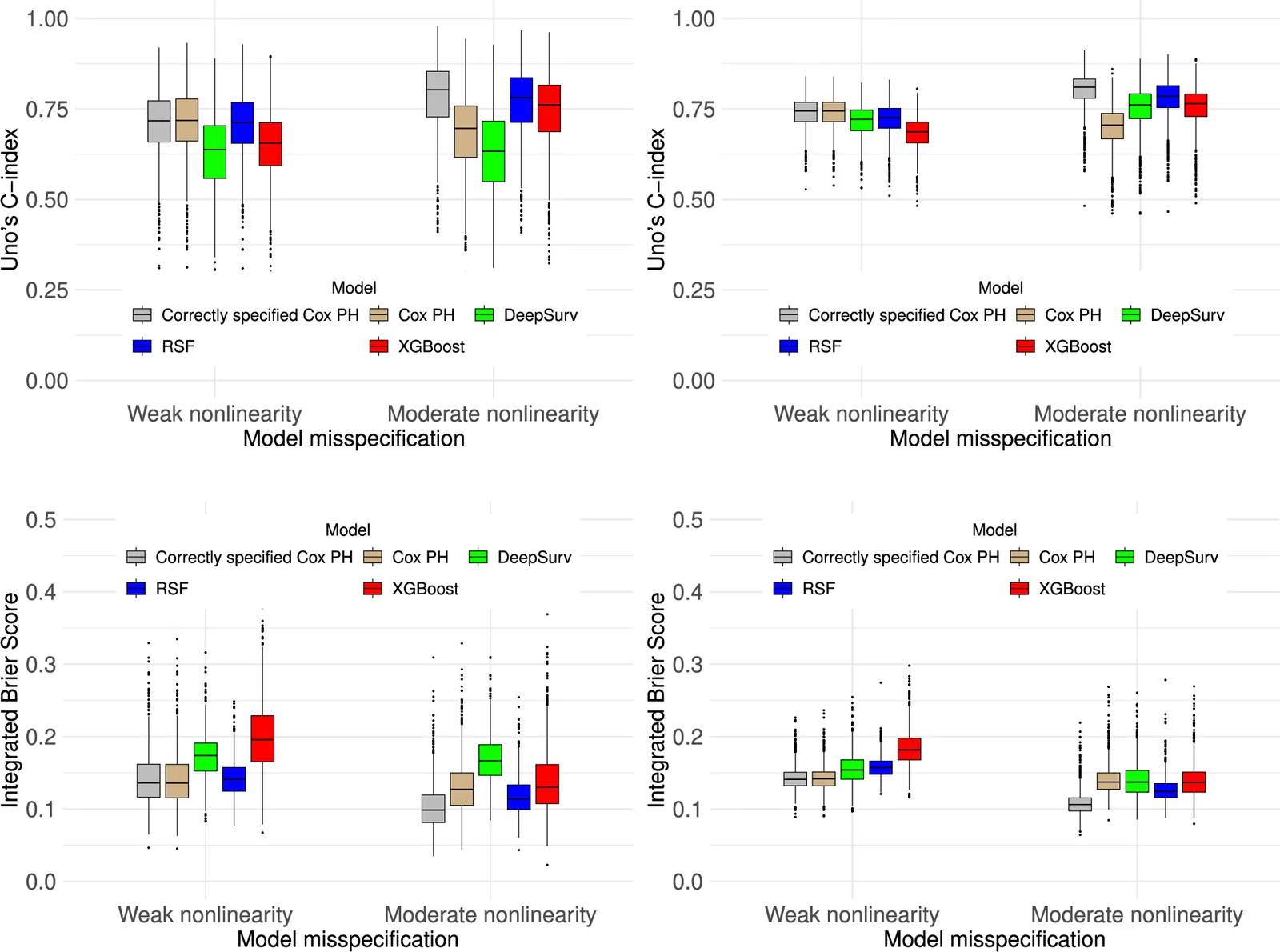
Prediction performance of the models with data-generating model misspecification, censoring proportion of 85% and sample sizes of 500 (left) and 5000 (right)

We assessed the performance of each model under different scenarios of model misspecification illustrated in Table 2, for both small (*n* = 500) and large (*n* = 5000) sample sizes. Fig. 4 displays the predictive performance for incorrectly specified models (non-linearity misspecification) compared to that of the correctly specified Cox PH model, based on data without censoring and with a 85% censoring proportion. The results are also summarized in Table B5 of Appendix B4. The findings suggest that, in the absence of either weak or moderate non-linearity misspecification in the Cox PH model, it performs well compared to both tree-based models and DeepSurv. On the other hand, in the presence of moderate non-linearity misspecification, tree-based models demonstrate more promising predictive performance compared to both the Cox PH model and DeepSurv.

### Stroke survival results

The results of the models evaluated on the real data (Riksstroke), based on various metrics, are presented in Table 4. All models performed well in the large sample real dataset. In line with the simulation results, DeepSurv demonstrated a survival prediction performance comparable to that of the Cox PH model in larger samples. All metrics indicated that XGBoost has the worst predictive performance when the sample size is small.

**Table 4:** Prediction performance of the models on the Riksstroke2015 − 2020 dataset

Model		Ischemic stroke			
	*n* = 70289	*n*_*r*_ = 10000		*n*_*r*_ = 500	
	*p* = 72%	*p* = 71%		*p* = 72%	
	Default	Default	Optimized Model	Default	Optimized Model
			**Harrell’s C-index**		
Cox PH	0.791 [0.785, 0.797]	0.782 [0.766, 0.797]	−	0.783 [0.720, 0.846]	−
DeepSurv	0.792 [0.786, 0.798]	0.783 [0.767, 0.798]	0.786 [0.771, 0.802]	0.746 [0.676, 0.816]	0.773 [0.707, 0.838]
XGBoost	0.790 [0.784, 0.796]	0.763 [0.747, 0.780]	0.783 [0.767, 0.798]	0.680 [0.593, 0.768]	0.715 [0.636, 0.793]
RSF	0.788 [0.782, 0.794]	0.782 [0.767, 0.798]	0.785 [0.770, 0.800]	0.754 [0.681, 0.828]	0.759 [0.674, 0.824]
			**Uno’s C-index**		
Cox PH	0.784 [0.777, 0.791]	0.777 [0.761, 0.792]	−	0.723 [0.629, 0.817]	−
DeepSurv	0.784 [0.778, 0.791]	0.776 [0.760, 0.791]	0.780 [0.764, 0.795]	0.689 [0.583, 0.795]	0.713 [0.612, 0.815]
XGBoost	0.781 [0.774, 0.788]	0.758 [0.741, 0.774]	0.778 [0.762, 0.794]	0.617 [0.514, 0.721]	0.670 [0.571, 0.769]
RSF	0.776 [0.769, 0.784]	0.773 [0.757, 0.789]	0.776 [0.760, 0.791]	0.711 [0.618, 0.803]	0.712 [0.619, 0.805]
			**Integrated Brier Score**		
Cox PH	0.133	0.138	−	0.153	−
DeepSurv	0.133	0.138	0.138	0.173	0.171
XGBoost	0.135	0.153	0.138	0.225	0.154
RSF	0.135	0.138	0.138	0.151	0.149

*n*: Sample size; *n*_*r*_: A random sample of *n*; *p*: Censoring proportion.

We also fitted the Cox PH model to the entire real dataset and assessed the PH assumption using the Schoenfeld residual test. The results revealed that the PH assumption in the Cox PH model was violated, with a *p*-value of  < 0.001 (Table C6 of Appendix C). On that note, the findings in Table 4 are comparable to the simulation results in Table B5 of Appendix B4, where the PH assumption was violated. The hyperparameter search space of the models and the optimized parameters from the random search tuning on the sub-samples from Riksstroke are displayed in Table 3. All other hyperparameters remained at their default values as shown in Table D7 of Appendix D. In both samples, model performance was generally similar, although hyperparameter tuning benefited XGBoost the most. In the larger sample size, the tuned ML models ranked higher than the Cox PH model in discrimination, with overlapping confidence intervals. The XGBoost model demonstrated improved performance on all evaluation measures, while the DeepSurv and RSF models showed only small improvements. In the smaller sample, tuning improved XGBoost and DeepSurv, while RSF demonstrated minimal improvement in terms of discrimination. Despite these changes, the Cox PH model maintained the highest discrimination, while the tuned RSF achieved the lowest overall prediction accuracy. In summary, hyperparameter tuning changed the ranking of Cox versus ML models in the larger sample but not in the smaller sample (Table 4).

## Discussion

This study has implemented a simulation framework to comprehensively evaluate and compare the performance of various ML methods with the traditional Cox PH model in predicting the risk of an event, that is, the time to death with an application to stroke survival. We selected the models most frequently used identified in the literature as having promising predictive performance [28]. The ML methods included two tree-based approaches (XGBoost for survival analysis and RSF) and a deep learning neural network (DeepSurv). For baseline comparisons, all models were trained using their default hyperparameters. Two model prediction evaluation metrics, C-index that accounts for the ranking of predictions, and IBS that measures model calibration, were utilized for model evaluation. The models were evaluated at the same follow-up interval for direct comparisons. The evaluation was based on simulated and real survival (Riksstroke) datasets. The simulated datasets were generated following different data-generating processes, with analyses accounting for factors such as varying sample size, censoring proportions, model misspecification, and inclusion of different numbers of noise variables. The Riksstroke dataset was used as an application example and not to demonstrate generalizability.

In agreement with previous studies that evaluated the survival prediction performance of the Cox model against ML methods, our results showed that the predictive performance of a model varies depending on the factors mentioned above [9, 14, 17, 18, 22, 56]. However, unlike these previous studies, this study conducted a more comprehensive evaluation by including a larger number of models, compared across a wider range of factors. Our findings indicated that the predictive performance of all models improved as the sample size increased. We also observed that as the sample size increases, the accuracy of DeepSurv becomes comparable to that of both the RSF and Cox PH models. This confirms that larger sample sizes increase the size of the risk set which improves the precision of hazard or survival estimates. From the analysis of the influence of censoring rates on prediction accuracy, all models showed a declining trend in predictive performance as censoring proportions increased, in terms of both Uno’s C-index and the IBS. Notably, the Cox PH model demonstrated a modest improvement in performance compared to the other models across all sample sizes and all levels of censoring.

As expected, the present study also found that the Cox model performed well, especially when the true model was correctly specified (either no non-linearity misspecification or no violation of PH assumption), and that was true for both small and large sample sizes. In contrast, tree-based models, specifically RSF and XGBoost, demonstrated a higher prediction performance than the Cox PH when the true model was misspecified. In survival analysis perspective, when the true hazard depends on non-linear covariate effects, the Cox PH model can perform poorly since they assume a log-linear relationship and proportional hazards. On the other hand, ML models are model-free and can flexibly estimate hazard functions without a predefined functional form.

All models showed unstable C-index values around 0.5, with wide confidence intervals, in the presence of many noise variables and a high level of censoring in a small dataset. This is an indication of weak or uncertain discrimination between risk levels, implying that these models may have limited practical predictive value in this scenario. In addition, noise variables introduce irrelevant information that increases variability hence increased risk of overfitting especially in the presence of high censoring. As censoring increased, the C-index remained relatively stable, whereas the IBS increased. This may be because the C-index primarily assesses the ordering of predicted risks and is less sensitive to errors in absolute survival probabilities, while the IBS evaluates prediction accuracy over time and therefore worsens when survival probabilities become increasingly miscalibrated.

The present results are consistent with the findings in the literature. A simulation study comparing the performance of methods to predict the time until conversion to mild cognitive impairment in different sample sizes concluded that the Cox PH model demonstrated a slight improvement in accuracy over both DeepSurv and RSF [14], which also aligns with the finding of Austin et al. [56]. The authors also noted that DeepSurv showed comparable accuracy in a larger sample size compared to the Cox PH model. The present study evaluated models with up to a larger sample size (*n* = 10000), however, stability in predictive performance was observed only in the Cox PH model, in terms of Uno’s C-index. Nonetheless, there was a minor difference in the performance of all the models up to the sample size mentioned, which is consistent with previous findings [9, 14]. Given that DeepSurv achieved only a modest improvement over the Cox PH model, its additional complexity and reduced interpretability may not be justified for routine stroke survival prediction when only structured data are available. The Cox PH model may be preferable for its interpretability and ease of implementation, provided that non-proportional hazards are accounted for.

A study evaluating the performance of ML methods (RSF and artificial neural networks) and the Cox regression model based on simulated time-to-event data highlighted that data censoring negatively impacted survival predictions for all the models. Furthermore, the ML methods outperformed the Cox regression model in predictive performance in the presence of non-linear model misspecification [22]. On a similar note, Golmakani and Polley pointed out that the basic Cox model performed best compared to the regularized Cox model and ML models in the presence of weak (they used smaller beta effects in the data generating model) non-linear misspecification in a low-dimensional dataset, which aligns with our findings as well [18].

Similarly to this study, the mentioned studies investigated the performance of the methods in different scenarios. However, we provided an extensive analysis that compared the prediction accuracy of the Cox PH model to that of ML methods by training the commonly applied models in the literature on the same datasets and across a broader range of scenarios. We included a larger number of covariates than was used in most of these studies [6, 9]. Additionally, we apply different performance metrics for the evaluation, including C-index which is based on ranking of predictions, and IBS.

Although this study offers valuable contributions, it is important to acknowledge some limitations that, although not necessarily influencing our findings, highlight areas for further exploration in future research. One limitation is that the covariates were generated independently without considering the correlation structure between them; however, in real datasets, many covariates are dependent. The parametric simulation design used in this study provides an analytically convenient hazard structure, allows controlled model comparisons, systematic variation of different factors mentioned earlier, and can serve as a fair benchmark [7, 51]. However, the design may favor the Cox PH model if the data generation process aligns with its assumptions [57] and potentially underrepresents the flexibility of ML models. Given the time-consuming nature of our simulation process, we opted to perform baseline comparisons using default hyperparameters for model training. However, this could lead to suboptimal performance of the ML models. Tuning can further improve the predictive performance of ML models. To address this, we complement the simulation study with an evaluation on a real dataset, where ML models are better suited to capture complex survival patterns. A random search to tune hyperparameters in the real dataset demonstrated a moderate increase in predictive performance when the sample size was small, and negligible or minor increase in larger samples. The simulation was based on Riksstroke dataset, which consisted mainly of categorical variables and a continuous variable. This may limit the generalizability of our findings to datasets with different structures or additional continuous variables. The IBS was calculated up to the maximum observed event time in each simulated dataset, ensuring fair model comparisons within iterations. However, because maximum times vary across iterations, the aggregated IBS estimates reflect slightly different time horizons, which may influence overall averages. Despite this, within-iteration comparisons remain valid, and the consistent relative performance of the models across most of the survival period supports the robustness of our findings. This study included Cox PH as the only conventional statistical model for comparison, which limits the evaluation of ML models against other statistical approaches. Future studies could consider relevant alternatives, such as accelerated failure time [58] and flexible parametric survival models [59]. The simulation included only structured predictors. However, in real-world settings, ML models may provide an additional advantage over conventional statistical models by integrating unstructured or multimodal data, such as medical images and electronic health records, with less manual feature engineering, as demonstrated in recent studies [60, 61].

## Conclusions

This study contributes to the field of survival analysis by comprehensively evaluating and comparing conventional models with ML models for predicting time-to-event data. We compare the accuracy of the most frequently used ML models with that of the Cox PH model, using both simulation studies under a wide range of data-generating mechanisms and real-world data. The findings reveal that the Cox PH model performs well, especially when the true model is specified correctly, and this holds true for both small and large sample sizes. Conversely, tree-based models showed better discrimination and overall predictive accuracy than the Cox PH model and deep neural networks (DeepSurv) in the presence of non-linear misspecification and a large number of noise variables. It is important that the appropriate model for a specific medical application is motivated by the research question and the underlying data structure. Each model has strengths depending on specific scenarios, such as when the model assumptions hold or not, the dataset size is small or large, when the censoring proportion is small or not, and the computational efficiency of the model. The ML models evaluated in this study may not have achieved their best possible performance and therefore require model-specific hyperparameter optimization in practice.

## Supplementary information

Supplementary Information

## Data Availability

The analysis code is publicly available in a Github repository https://github.com/Josline90/Cox-vs-ML-models-for-time-to-event-data_A-simulation-study.git. The real data analyzed in the study consist of third-party data from Riksstroke and its use has been approved by the Swedish Ethical Review Authority (reference number 2021 - 06152 - 01). According to Swedish legislation data cannot be made available for use beyond what has been approved by the ethical review authority. Therefore, the data cannot be made publicly available. Data may be made available from Riksstroke (contact via riksstroke@regionvasterbotten.se) upon reasonable request by researchers who meet the criteria for access to confidential data according to Swedish laws and regulations.
