# Supplementary material for: A comprehensive simulation study evaluating the predictive performance of Cox proportional hazards model and machine learning methods for time-to-event data
Source: BMC Med Res Methodol. 2026 Nov 10;26:240. doi: 10.1186/s12874-026-03050-5 (PMC13749909; doi:10.1186/s12874-026-03050-5)
Supplement: Supplementary file 1 [file 12874_2026_3050_MOESM1_ESM.pdf]

A comprehensive simulation study evaluating the predictive performance of Cox proportional hazards model and machine learning methods for time-to-event data

Josline Adhiambo Otieno[1*], Jenny Häggström[1] and Marie Eriksson[1]

[1*]Department of Statistics, USBE, Umeå University, Samhällsvetarhuset, Biblioteksgränd 6, Umeå, 901 87, Sweden.

*Corresponding author(s). E-mail(s): josline.otieno@umu.se; Contributing authors: jenny.haggstrom@umu.se; marie.eriksson@umu.se;

Appendix A Simulating covariates for survival data

Table A1 Simulating data for the study.

**Covariate** (X_i)	**Distribution**	**Coefficient** (β_i)
x_{i1}	`rtruncnorm(n = n, a = 18, b = 108, mean = 74.36, sd = 12.44)`	0.810
x_{i2}	`rbinom(n = n, size = 1, prob = 0.55)`	0.242
x_{i3}	`rbinom(n = n, size = 1, prob = 0.27)`	0.311
x_{i4}	`rbinom(n = n, size = 1, prob = 0.22)`	0.269
x_{i5}	`rbinom(n = n, size = 1, prob = 0.27)`	0.086
x_{i6}	`rbinom(n = n, size = 1, prob = 0.14)`	0.269
x_{i7}	`rbinom(n = n, size = 1, prob = 0.62)`	0.027
x_{i8}	`rbinom(n = n, size = 1, prob = 0.32)`	-0.060
x_{i9}	`rbinom(n = n, size = 1, prob = 0.13)`	-0.037
x_{i10}	`rbinom(n = n, size = 1, prob = 0.25)`	0.870
(x_{i11}, x_{i12})	`apply(rmultinom(n = n, size = 1, prob = c(0.91, 0.07, 0.02)), 2, which.max)`	(0.968, 1.819)
x_{i13}	`rbinom(n = n, size = 1, prob = 0.04)`	0.188
x_{i14}	`rbinom(n = n, size = 1, prob = 0.15)`	-0.201
x_{i15}	`rbinom(n = n, size = 1, prob = 0.23)`	-0.044
x_{i16}	`rbinom(n = n, size = 1, prob = 0.85)`	-0.088
x_{i17}	`rbinom(n = n, size = 1, prob = 0.39)`	0.094
x_{i18}	`rbinom(n = n, size = 1, prob = 0.73)`	0.440

Appendix B Data generating mechanisms

B.1 Varying sample sizes

Table B2 Predictive performance for varying sample sizes (17 covariates, censoring proportion of 85%).

Model	Sample size			
	500	1000	5000	10000
**Harrell's C-index**				
Cox PH	0.719 [0.601, 0.837]	0.740 [0.659, 0.821]	0.755 [0.719, 0.790]	0.757 [0.732, 0.782]
DeepSurv	0.637 [0.510, 0.763]	0.669 [0.581, 0.758]	0.730 [0.693, 0.767]	0.744 [0.719, 0.770]
XGBoost	0.664 [0.540, 0.788]	0.672 [0.584, 0.760]	0.700 [0.662, 0.738]	0.715 [0.688, 0.741]
RSF	0.717 [0.600, 0.835]	0.726 [0.642, 0.809]	0.740 [0.703, 0.777]	0.743 [0.717, 0.769]
**Uno's C-index**				
Cox PH	0.701 [0.542, 0.861]	0.719 [0.591, 0.847]	0.734 [0.657, 0.812]	0.734 [0.669, 0.802]
DeepSurv	0.620 [0.449, 0.791]	0.653 [0.516, 0.791]	0.711 [0.631, 0.791]	0.721 [0.651, 0.790]
XGBoost	0.641 [0.469, 0.813]	0.653 [0.516, 0.791]	0.677 [0.593, 0.761]	0.689 [0.617, 0.761]
RSF	0.696 [0.533, 0.859]	0.703 [0.571, 0.836]	0.715 [0.634, 0.796]	0.717 [0.647, 0.786]
**Integrated Brier Score**				
Cox PH	0.143	0.142	0.145	0.147
DeepSurv	0.172	0.172	0.157	0.155
XGBoost	0.206	0.208	0.189	0.178
RSF	0.144	0.151	0.161	0.166

B.2 Varying censoring proportions

Table B3 Prediction performances of the models for varying censoring proportions (17 covariates, sample sizes of 500, 1000, and 5000).

Model	Sample size = 500				
	**Censoring proportion**				
	0%	10%	50%	70%	85%
			**Harrell's C-index**		
Cox PH	0.720 [0.676, 0.764]	0.722 [0.676, 0.768]	0.729 [0.668, 0.791]	0.731 [0.651, 0.811]	0.719 [0.601, 0.837]
DeepSurv	0.624 [0.573, 0.674]	0.626 [0.574, 0.679]	0.635 [0.566, 0.704]	0.638 [0.549, 0.727]	0.637 [0.510, 0.763]
XGBoost	0.670 [0.622, 0.718]	0.670 [0.620, 0.720]	0.673 [0.606, 0.739]	0.670 [0.584, 0.756]	0.664 [0.540, 0.788]
RSF	0.702 [0.657, 0.746]	0.704 [0.657, 0.750]	0.714 [0.651, 0.777]	0.719 [0.637, 0.801]	0.717 [0.600, 0.835]
			**Uno's C-index**		
Cox PH	0.720 [0.676, 0.764]	0.720 [0.676, 0.764]	0.716 [0.654, 0.777]	0.711 [0.611, 0.810]	0.701 [0.542, 0.861]
DeepSurv	0.624 [0.573, 0.674]	0.625 [0.574, 0.676]	0.626 [0.558, 0.693]	0.625 [0.517, 0.732]	0.620 [0.449, 0.791]
XGBoost	0.670 [0.622, 0.718]	0.668 [0.619, 0.716]	0.658 [0.592, 0.725]	0.653 [0.547, 0.758]	0.641 [0.469, 0.813]
RSF	0.702 [0.657, 0.746]	0.702 [0.657, 0.746]	0.699 [0.637, 0.762]	0.697 [0.595, 0.799]	0.696 [0.533, 0.859]
			**Integrated Brier Score**		
Cox PH	0.030	0.053	0.137	0.164	0.143
DeepSurv	0.037	0.057	0.145	0.181	0.172
XGBoost	0.054	0.092	0.223	0.260	0.206
RSF	0.034	0.058	0.149	0.176	0.144
			Sample size = 1000		
			**Harrell's C-index**		
Cox PH	0.723 [0.693, 0.754]	0.725 [0.693, 0.757]	0.735 [0.692, 0.778]	0.741 [0.685, 0.797]	0.740 [0.659, 0.821]
DeepSurv	0.656 [0.622, 0.690]	0.658 [0.622, 0.693]	0.667 [0.620, 0.714]	0.671 [0.610, 0.732]	0.669 [0.581, 0.758]
XGBoost	0.680 [0.647, 0.712]	0.681 [0.646, 0.715]	0.682 [0.636, 0.729]	0.681 [0.620, 0.741]	0.672 [0.584, 0.760]
RSF	0.708 [0.677, 0.738]	0.710 [0.677, 0.742]	0.721 [0.677, 0.765]	0.726 [0.669, 0.783]	0.726 [0.642, 0.809]
			**Uno's C-index**		
Cox PH	0.723 [0.693, 0.754]	0.723 [0.692, 0.753]	0.722 [0.679, 0.764]	0.723 [0.651, 0.795]	0.719 [0.591, 0.847]
DeepSurv	0.656 [0.622, 0.690]	0.656 [0.621, 0.690]	0.656 [0.610, 0.702]	0.656 [0.579, 0.734]	0.653 [0.516, 0.791]
XGBoost	0.680 [0.647, 0.712]	0.678 [0.645, 0.712]	0.668 [0.623, 0.714]	0.662 [0.584, 0.739]	0.653 [0.516, 0.791]
RSF	0.708 [0.677, 0.738]	0.707 [0.676, 0.739]	0.706 [0.663, 0.749]	0.707 [0.633, 0.781]	0.703 [0.571, 0.836]
			**Integrated Brier Score**		
Cox PH	0.024	0.044	0.127	0.157	0.142
DeepSurv	0.028	0.046	0.130	0.172	0.172
XGBoost	0.037	0.067	0.188	0.235	0.208
RSF	0.026	0.048	0.139	0.172	0.151
			Sample size = 5000		
			**Harrell's C-index**		
Cox PH	0.726 [0.713, 0.740]	0.728 [0.714, 0.742]	0.739 [0.720, 0.758]	0.747 [0.723, 0.772]	0.755 [0.719, 0.790]
DeepSurv	0.708 [0.695, 0.722]	0.710 [0.695, 0.725]	0.720 [0.700, 0.739]	0.726 [0.701, 0.751]	0.730 [0.693, 0.767]
XGBoost	0.701 [0.687, 0.715]	0.702 [0.687, 0.717]	0.708 [0.688, 0.728]	0.707 [0.681, 0.733]	0.700 [0.662, 0.738]
RSF	0.717 [0.704, 0.731]	0.719 [0.705, 0.733]	0.730 [0.711, 0.749]	0.736 [0.711, 0.761]	0.740 [0.703, 0.777]
			**Uno's C-index**		
Cox PH	0.726 [0.713, 0.740]	0.726 [0.712, 0.739]	0.726 [0.707, 0.744]	0.728 [0.691, 0.765]	0.734 [0.657, 0.812]
DeepSurv	0.708 [0.695, 0.722]	0.708 [0.694, 0.722]	0.707 [0.688, 0.726]	0.707 [0.669, 0.746]	0.711 [0.631, 0.791]
XGBoost	0.701 [0.687, 0.715]	0.700 [0.685, 0.714]	0.692 [0.672, 0.712]	0.684 [0.644, 0.724]	0.677 [0.593, 0.761]
RSF	0.717 [0.704, 0.731]	0.717 [0.703, 0.730]	0.714 [0.696, 0.733]	0.713 [0.674, 0.752]	0.715 [0.634, 0.796]
			**Integrated Brier Score**		
Cox PH	0.015	0.032	0.110	0.149	0.145
Deepsurv	0.015	0.029	0.101	0.144	0.157
XGBoost	0.018	0.038	0.133	0.186	0.189
RSF	0.016	0.034	0.121	0.167	0.161

B.3 Varying number of noise variables

Table B4 Prediction performances of the models for varying number of noise variables (17 covariates, censoring proportion of 85%).

**Model**	Sample size = 500			
	**Number of noise variables**			
	0	15	100	500
	**Harrell's C-index**			
Cox PH	0.719 [0.601, 0.837]	0.694 [0.572, 0.815]	0.583 [0.451, 0.715]	0.502 [0.368, 0.636]
DeepSurv	0.637 [0.510, 0.763]	0.578 [0.447, 0.710]	0.533 [0.398, 0.668]	0.514 [0.380, 0.649]
XGBoost	0.664 [0.540, 0.788]	0.657 [0.533, 0.781]	0.631 [0.504, 0.759]	0.605 [0.474, 0.735]
RSF	0.717 [0.600, 0.835]	0.703 [0.582, 0.823]	0.672 [0.546, 0.797]	0.626 [0.495, 0.757]
	**Uno's C-index**			
Cox PH	0.701 [0.542, 0.861]	0.673 [0.505, 0.840]	0.576 [0.398, 0.753]	0.499 [0.323, 0.676]
DeepSurv	0.620 [0.449, 0.791]	0.568 [0.391, 0.744]	0.527 [0.348, 0.706]	0.512 [0.332, 0.692]
XGBoost	0.641 [0.469, 0.813]	0.641 [0.470, 0.813]	0.624 [0.452, 0.795]	0.596 [0.422, 0.770]
RSF	0.696 [0.533, 0.859]	0.675 [0.506, 0.844]	0.651 [0.480, 0.822]	0.614 [0.440, 0.788]
	**Integrated Brier Score**			
Cox PH	0.143	0.162	0.306	0.459
DeepSurv	0.172	0.180	0.186	0.187
XGBoost	0.206	0.208	0.281	0.501
RSF	0.144	0.151	0.156	0.161
	Sample size = 1000			
	**Harrell's C-index**			
Cox PH	0.740 [0.659, 0.821]	0.725 [0.643, 0.808]	0.655 [0.565, 0.745]	0.539 [0.444, 0.635]
DeepSurv	0.669 [0.581, 0.758]	0.614 [0.523, 0.706]	0.551 [0.456, 0.646]	0.525 [0.430, 0.621]
XGBoost	0.672 [0.584, 0.760]	0.674 [0.587, 0.761]	0.662 [0.574, 0.750]	0.640 [0.551, 0.729]
RSF	0.726 [0.642, 0.809]	0.719 [0.635, 0.804]	0.698 [0.610, 0.786]	0.659 [0.568, 0.751]
	**Uno's C-index**			
Cox PH	0.719 [0.591, 0.847]	0.710 [0.584, 0.837]	0.635 [0.495, 0.774]	0.534 [0.390, 0.678]
DeepSurv	0.653 [0.516, 0.791]	0.603 [0.460, 0.746]	0.548 [0.402, 0.694]	0.526 [0.382, 0.670]
XGBoost	0.653 [0.516, 0.791]	0.658 [0.522, 0.794]	0.646 [0.508, 0.783]	0.630 [0.494, 0.766]
RSF	0.703 [0.571, 0.836]	0.699 [0.569, 0.830]	0.671 [0.532, 0.810]	0.634 [0.496, 0.773]
	**Integrated Brier Score**			
Cox PH	0.142	0.150	0.213	0.445
DeepSurv	0.172	0.181	0.186	0.192
XGBoost	0.208	0.210	0.222	0.240
RSF	0.151	0.156	0.164	0.172
	Sample size = 5000			
	**Harrell's C-index**			
Cox PH	0.755 [0.719, 0.790]	0.751 [0.716, 0.787]	0.735 [0.699, 0.772]	0.666 [0.627, 0.706]
DeepSurv	0.730 [0.693, 0.767]	0.707 [0.669, 0.745]	0.649 [0.609, 0.689]	0.560 [0.517, 0.602]
XGBoost	0.700 [0.662, 0.738]	0.696 [0.658, 0.735]	0.696 [0.657, 0.734]	0.689 [0.651, 0.728]
RSF	0.740 [0.703, 0.777]	0.739 [0.702, 0.776]	0.732 [0.695, 0.770]	0.714 [0.675, 0.753]
	**Uno's C-index**			
Cox PH	0.734 [0.657, 0.812]	0.732 [0.653, 0.811]	0.710 [0.628, 0.792]	0.644 [0.557, 0.731]
DeepSurv	0.711 [0.631, 0.791]	0.688 [0.603, 0.773]	0.632 [0.544, 0.720]	0.552 [0.461, 0.643]
XGBoost	0.677 [0.593, 0.761]	0.675 [0.590, 0.761]	0.672 [0.586, 0.758]	0.668 [0.582, 0.754]
RSF	0.715 [0.634, 0.796]	0.716 [0.633, 0.798]	0.705 [0.621, 0.789]	0.681 [0.594, 0.769]
	**Integrated Brier Score**			
Cox PH	0.145	0.147	0.155	0.216
DeepSurv	0.157	0.166	0.179	0.197
XGBoost	0.189	0.195	0.201	0.233
RSF	0.161	0.165	0.174	0.184

B.4 Model misspecification

Table B5 Prediction performances of the models with data generating model misspecification and sample sizes of 500 and 5000 (17 covariates).

Model	Model Specification					
	Weak non-linearity	Moderate non-linearity	Violated PH assumption	Weak non-linearity	Moderate non-linearity	Violated PH assumption
	**0**% censoring proportion			**85**% censoring proportion		
Sample size = 500						
**Harrell's C-index**						
Correctly specified Cox PH	0.720 [0.676, 0.764]	0.751 [0.708, 0.793]	0.798 [0.759, 0.836]	0.732 [0.616, 0.849]	0.855 [0.763, 0.946]	0.775 [0.661, 0.890]
Misspecified Cox PH	0.719 [0.675, 0.763]	0.661 [0.609, 0.713]	0.780 [0.743, 0.817]	0.735 [0.619, 0.850]	0.741 [0.600, 0.881]	0.732 [0.616, 0.847]
DeepSurv	0.623 [0.572, 0.673]	0.587 [0.534, 0.641]	0.623 [0.573, 0.674]	0.649 [0.525, 0.774]	0.681 [0.552, 0.811]	0.629 [0.504, 0.755]
XGBoost	0.668 [0.621, 0.716]	0.708 [0.662, 0.755]	0.738 [0.697, 0.778]	0.679 [0.557, 0.801]	0.826 [0.727, 0.924]	0.683 [0.561, 0.805]
RSF	0.703 [0.658, 0.747]	0.715 [0.670, 0.760]	0.761 [0.724, 0.798]	0.734 [0.619, 0.849]	0.839 [0.745, 0.932]	0.724 [0.610, 0.839]
**Uno's C-index**						
Correctly specified Cox PH	0.720 [0.676, 0.764]	0.751 [0.708, 0.793]	0.798 [0.759, 0.836]	0.709 [0.552, 0.866]	0.785 [0.633, 0.937]	–
Misspecified Cox PH	0.719 [0.675, 0.763]	0.661 [0.609, 0.713]	0.780 [0.743, 0.817]	0.712 [0.557, 0.867]	0.684 [0.500, 0.868]	0.768 [0.637, 0.899]
DeepSurv	0.623 [0.572, 0.673]	0.587 [0.534, 0.641]	0.623 [0.573, 0.674]	0.627 [0.458, 0.797]	0.627 [0.446, 0.807]	0.623 [0.465, 0.780]
XGBoost	0.668 [0.621, 0.716]	0.708 [0.662, 0.755]	0.738 [0.697, 0.778]	0.648 [0.478, 0.818]	0.744 [0.580, 0.908]	0.704 [0.562, 0.847]
RSF	0.703 [0.658, 0.747]	0.715 [0.670, 0.760]	0.761 [0.724, 0.798]	0.705 [0.545, 0.866]	0.767 [0.607, 0.926]	0.782 [0.656, 0.907]
**Integrated Brier Score**						
Correctly specified Cox PH	0.038	0.044	–	0.142	0.103	–
Misspecified Cox PH	0.039	0.057	0.025	0.141	0.131	0.122
DeepSurv	0.045	0.055	0.034	0.173	0.169	0.177
XGBoost	0.064	0.069	0.051	0.201	0.138	0.194
RSF	0.041	0.049	0.029	0.143	0.117	0.119
Sample size = 5000						
**Harrell's C-index**						
Correctly specified Cox PH	0.725 [0.712, 0.739]	0.757 [0.744, 0.770]	0.801 [0.789, 0.812]	0.767 [0.732, 0.801]	0.878 [0.851, 0.904]	0.804 [0.769, 0.839]
Misspecified Cox PH	0.724 [0.711, 0.738]	0.671 [0.655, 0.687]	0.783 [0.772, 0.794]	0.766 [0.731, 0.801]	0.773 [0.726, 0.819]	0.755 [0.720, 0.791]
DeepSurv	0.707 [0.693, 0.721]	0.706 [0.693, 0.720]	0.753 [0.741, 0.764]	0.744 [0.707, 0.780]	0.839 [0.808, 0.870]	0.733 [0.697, 0.770]
XGBoost	0.701 [0.687, 0.715]	0.740 [0.726, 0.754]	0.766 [0.754, 0.778]	0.715 [0.677, 0.753]	0.851 [0.821, 0.881]	0.719 [0.681, 0.757]
RSF	0.717 [0.704, 0.730]	0.740 [0.726, 0.753]	0.772 [0.761, 0.784]	0.753 [0.717, 0.789]	0.864 [0.836, 0.892]	0.739 [0.702, 0.776]
**Uno's C-index**						
Correctly specified Cox PH	0.725 [0.712, 0.739]	0.757 [0.744, 0.770]	0.801 [0.789, 0.812]	0.739 [0.662, 0.816]	0.801 [0.716, 0.885]	–
Misspecified Cox PH	0.724 [0.711, 0.738]	0.671 [0.655, 0.687]	0.783 [0.772, 0.794]	0.739 [0.661, 0.816]	0.699 [0.594, 0.804]	0.800 [0.740, 0.860]
DeepSurv	0.707 [0.693, 0.721]	0.706 [0.693, 0.720]	0.753 [0.741, 0.764]	0.716 [0.637, 0.796]	0.751 [0.655, 0.847]	0.767 [0.702, 0.832]
XGBoost	0.701 [0.687, 0.715]	0.740 [0.726, 0.754]	0.766 [0.754, 0.778]	0.683 [0.600, 0.766]	0.755 [0.659, 0.850]	0.742 [0.675, 0.809]
RSF	0.717 [0.704, 0.730]	0.740 [0.726, 0.753]	0.772 [0.761, 0.784]	0.721 [0.640, 0.802]	0.778 [0.684, 0.871]	0.787 [0.723, 0.851]
**Integrated Brier Score**						
Correctly specified Cox PH	0.022	0.027	–	0.142	0.108	–
Misspecified Cox PH	0.022	0.035	0.013	0.143	0.141	0.115
DeepSurv	0.022	0.029	0.013	0.156	0.140	0.135
XGBoost	0.025	0.031	0.015	0.184	0.139	0.153
RSF	0.023	0.029	0.014	0.158	0.127	0.123

Appendix C Cox PH analysis for 2015 − 2020 Riksstroke data set

Table C6 displays the parameter estimates from the Cox proportional hazards model fitted to the entire Swedish Stroke registry dataset used in this study. The hazard ratios indicate the effect of each covariate on the risk of death in stroke patients. Values greater than 1 show an increased risk, while values less than 1 show a decreased risk of death. The $p-$values test the null hypothesis of no association between the covariate and the risk of death, that is, the hazard ratio for the covariate is 1. A $p-$value < 0.05 suggests statistical significance. We also display the summary statistics, including the mean and standard deviation for the continuous variable and the proportion (in percentage) for binary and categorical variables. These statistics represent the parameter values used to sample the variables from different distributions in our simulation. Additionally, the model coefficients are shown, corresponding to the betas used in generating the datasets for this study.

Table C6 Estimates from Cox PH analysis for $2015-2020$ Riksstroke data set (70289 ischemic stroke patients).

#	Variable		Coefficient (β_i)	Hazard ratio (e^{β_i})	95% CI for e^{β_i}	$p-$value	p-value Schoenfeld
1	Age (mean [sd])	74.36 [12.44]	0.810*	2.249	[2.198, 2.301]	<0.001	<0.001
2	Male (%)	38985 (55.46)	0.242	1.274	[1.237, 1.313]	<0.001	<0.001
3	Atrial fibrillation (%)	18839 (26.8)	0.311	1.365	[1.320, 1.412]	<0.001	0.352
4	Diabetes (%)	15532 (22.1)	0.269	1.309	[1.266, 1.354]	<0.001	<0.001
5	Previous stroke (TIA) (%)	18808 (26.76)	0.086	1.089	[1.056, 1.124]	<0.001	<0.001
6	Smoking (%)	10086 (14.35)	0.269	1.309	[1.246, 1.375]	<0.001	0.950
7	Hypertension (%)	43794 (62.31)	0.027	1.027	[0.995, 1.061]	0.10	<0.001
8	Lipid-lowering medication (%)	22770 (32.39)	-0.060	0.942	[0.911, 0.973]	<0.001	<0.001
9	Prior-anticoagulation (%)	9137 (13)	-0.037	0.964	[0.923, 1.005]	0.088	<0.001
10	mRS prior to the stroke $(3-5)$ (%)	17448 (24.82)	0.870	2.387	[2.311, 2.466]	<0.001	0.778
	Loss of consciousness (%)						
	1 (alert) (*reference*)	64014 (91.07)					
11	2 (drowsy)	4887 (6.95)	0.968	2.632	[2.527, 2.742]	<0.001	<0.001
12	3 (unconscious)	1075 (1.53)	1.819	6.165	[5.741, 6.621]	<0.001	<0.001
13	Thrombectomy (%)	2752 (3.92)	0.188	1.206	[1.117, 1.303]	<0.001	<0.001
14	Thrombolysis (%)	10540 (15)	-0.201	0.818	[0.780, 0.859]	<0.001	<0.001
15	Wake up stroke (%)	16252 (23.12)	-0.044	0.957	[0.925, 0.991]	0.012	0.064
16	Admission at stroke unit (%)	59716 (84.96)	-0.088	0.916	[0.881, 0.952]	<0.001	0.337
17	Stroke alert activation (%)	27065 (38.51)	0.094	1.099	[1.062, 1.137]	<0.001	<0.001
18	Ambulance (%)	51259 (72.93)	0.440	1.552	[1.485, 1.623]	<0.001	<0.001
				Global $p-$**value** for Schoenfeld residual test			<0.001

Since predictor age was rescaled, * indicates the change in the log hazard ratio for a one standard deviation increase in age.

Appendix D Hyperparameter tuning

Table D7: Model hyperparameters search space and optimized parameters from random search tuning, based on 2015 – 2020 Riksstroke data set (ischemic stroke patients).

Hyperparameter		Default	Search space	Optimized parameters	
				Sample size: 10000	Sample size: 500
**DeepSurv**					
num_nodes	num_layers	2	$[5, 20]$	20	12
	num_nodes_per_layer	32	$[10, 50]$	15	35
dropout		_	$[0, 0.5]$	0.212	0.086
weight_decay		0	$[0, 0.5]$	0.079	0.227
learning_rate		0.01	$[0, 0.05]$	0.030	0.042
activation		"relu"	$c($"*relu*", "*sigmoid*"$)$	"*relu*"	"*relu*"
epochs		1	$[1, 500]$	13	387
Default and/ User-Defined					
early_stopping		FALSE	_	TRUE	TRUE
optimizer		"*adam*"	_	"*adam*"	"*adam*"
frac		0	_	0	0
batch_norm		TRUE	_	TRUE	TRUE
best_weights		FALSE	_	FALSE	FALSE
min_delta		0	_	0	0
patience		10	_	10	10
lambd		0.0001	_	0.0001	0.0001
batch_size		256	_	256	256
nesterov		FALSE	_	FALSE	FALSE
rho		0.9	_	0.9	0.9
num_workers		0	_	0	0
lr_decay		0	_	0	0
shuffle		TRUE	_	TRUE	TRUE
eps		0.00000001	_	0.00000001	0.00000001
verbose		TRUE	_	TRUE	TRUE
lr		1	_	1	1
betas		$c(0.9, 0.999)$	_	$c(0.9, 0.999)$	$c(0.9, 0.999)$
amsgrad		FALSE	_	FALSE	FALSE
alpha		0.75	_	0.75	0.75
t0		1000000	_	1000000	1000000
momentum		0	_	0	0
centered		TRUE	_	TRUE	TRUE
etas		$c(0.5, 1.2)$	_	$c(0.5, 1.2)$	$c(0.5, 1.2)$
step_sizes		$c(0.000001, 50)$	_	$c(0.000001, 50)$	$c(0.000001, 50)$
dampening		0	_	0	0
device		NULL	_	NULL	NULL
**XGBoost**					
alpha		0	$[0, 10]$	9.652	7.552
subsample		1	$[0.5, 1]$	0.973	0.574
nrounds		_	$[50, 1000]$	540	158
colsample_bytree		1	$[0, 1]$	0.145	0.044
max_depth		6	$[3, 40]$	23	38
eta		0.3	$[0.0001, 1]$	0.054	0.313
gamma		0	$[0.0001, 1]$	0.666	0.468
lambda		1	$[0, 10]$	9.570	1.717
min_child_weight		1	$[1, 10]$	3	2
Default					
colsample_bylevel		1	_	1	1
colsample_bynode		1	_	1	1
booster		"gbtree"	_	"gbtree"	"gbtree"
callbacks		list()	_	list()	list()
disable_default_eval_metric		FALSE	_	FALSE	FALSE
early_stopping_rounds		NULL	_	NULL	NULL
feature_selector		cyclic	_	cyclic	cyclic
feval		NULL	_	NULL	NULL

Hyperparameter	Default	Search space	Optimized parameters	
			Sample size: 10000	Sample size: 500
grow_policy	depthwise	-	depthwise	depthwise
interaction_constraints	-	-	-	-
iterationrange	-	-	-	-
lambda_bias	0	-	0	0
max_bin	256	-	256	256
max_delta_step	0	-	0	0
max_leaves	0	-	0	0
maximize	NULL	-	NULL	NULL
missing	NA	-	NA	NA
monotone_constraints	0	-	0	0
normalize_type	tree	-	tree	tree
nthread	1	-	1	1
num_parallel_tree	1	-	1	1
one_drop	FALSE	-	FALSE	FALSE
print_every_n	1	-	1	1
process_type	default	-	default	default
rate_drop	0	-	0	0
refresh_leaf	TRUE	-	TRUE	TRUE
save_name	-	-	-	-
sampling_method	uniform	-	uniform	uniform
save_period	-	-	-	-
scale_pos_weight	1	-	1	1
seed_per_iteration	FALSE	-	FALSE	FALSE
skip_drop	0	-	0	0
strict_shape	FALSE	-	FALSE	FALSE
top_k	0	-	0	0
tweedie_variance_power	1.5	-	1.5	1.5
updater	-	-	-	-
verbose	1	-	1	1
watchlist	NULL	-	NULL	NULL
xgb_model	-	-	-	-
device	-	-	-	-
base_score	0.5	-	0.5	0.5
tree_method	auto	-	auto	auto
sample_type	uniform	-	uniform	uniform
		**RSF**		
nsplit	10	$[10, 20]$	16	19
ntree	500	$[400, 1000]$	689	551
mtry	$\sqrt{p}$	$[1, 20]$	3	3
nodesize	15	$[5, 20]$	19	9
nodedepth	-	$[10, 100]$	12	59
Default				
case.depth	FALSE	-	FALSE	FALSE
splitrule	logrank	-	logrank	logrank
importance	FALSE ()	FALSE	FALSE	
block.size	10	-	10	10
bootstrap	by.root	-	by.root	by.root
samptype	swor	-	swor	swor
samp	-	-	-	-
membership	FALSE	-	FALSE	FALSE
sampsize	-	-	-	-
mtry.ratio	-	-	-	-
sampsize.ratio	-	-	-	-
na.action	na.omit	-	na.omit	na.omit
nimpute	1	-	1	1
ntime	150	-	150	150
cause	-	-	-	-
proximity	FALSE	-	FALSE	FALSE

Hyperparameter	Default	Search space	Optimized parameters	
			Sample size: 10000	Sample size: 500
distance	FALSE	–	FALSE	FALSE
forest.wt	FALSE	–	FALSE	FALSE
xvar.wt	–	–	–	–
split.wt	–	–	–	–
forest	TRUE	–	TRUE	TRUE
var.used	FALSE	–	FALSE	FALSE
split.depth	FALSE	–	FALSE	FALSE
do.trace	FALSE	–	FALSE	FALSE
statistics	FALSE	–	FALSE	FALSE
estimator	nelson	–	nelson	nelson
get.tree	–	–	–	–
outcome	train	–	train	train
ptn.count	0	–	0	0
cores	1	–	1	1
save.memory	FALSE	–	FALSE	FALSE
perf.type	–	–	–	–
case.depth	FALSE	–	FALSE	FALSE
seed	–	–	–	–
